# Cell envelope stress in mycobacteria is regulated by the novel signal transduction ATPase IniR in response to trehalose

**DOI:** 10.1371/journal.pgen.1007131

**Published:** 2017-12-27

**Authors:** Maikel Boot, Vincent J. C. van Winden, Marion Sparrius, Robert van de Weerd, Alexander Speer, Roy Ummels, Tige Rustad, David R. Sherman, Wilbert Bitter

**Affiliations:** 1 Department of Medical Microbiology and Infection Control, VU University Medical Center, Amsterdam, the Netherlands; 2 Center for Infectious Disease, Seattle, Washington, United States of America; 3 Department of Molecular Microbiology, VU University, Amsterdam, the Netherlands; Institut Pasteur, CNRS UMR 3525, FRANCE

## Abstract

The cell envelope of mycobacteria is a highly unique and complex structure that is functionally equivalent to that of Gram-negative bacteria to protect the bacterial cell. Defects in the integrity or assembly of this cell envelope must be sensed to allow the induction of stress response systems. The promoter that is specifically and most strongly induced upon exposure to ethambutol and isoniazid, first line drugs that affect cell envelope biogenesis, is the *iniBAC* promoter. In this study, we set out to identify the regulator of the *iniBAC* operon in *Mycobacterium marinum* using an unbiased transposon mutagenesis screen in a constitutively *iniBAC*-expressing mutant background. We obtained multiple mutants in the *mce1* locus as well as mutants in an uncharacterized putative transcriptional regulator (*MMAR_0612*). This latter gene was shown to function as the *iniBAC* regulator, as overexpression resulted in constitutive *iniBAC* induction, whereas a knockout mutant was unable to respond to the presence of ethambutol and isoniazid. Experiments with the *M*. *tuberculosis* homologue (*Rv0339c*) showed identical results. RNAseq experiments showed that this regulatory gene was exclusively involved in the regulation of the *iniBAC* operon. We therefore propose to name this dedicated regulator ***ini****BAC* **R**egulator (IniR). IniR belongs to the family of signal transduction ATPases with numerous domains, including a putative sugar-binding domain. Upon testing different sugars, we identified trehalose as an activator and metabolic cue for *iniBAC* activation, which could also explain the effect of the *mce1* mutations. In conclusion, cell envelope stress in mycobacteria is regulated by IniR in a cascade that includes trehalose.

## Author summary

The mycobacterial cell wall is a complex and unique structure that protects extremely well against harmful compounds. Understanding the biogenesis and functioning of this cell envelope is essential to be able to effectively target mycobacteria. One way to uncover cell envelope functionality is to study stress mechanisms that are induced when the cell envelope is damaged. Here, we describe the identification of a major cell envelope stress regulator and the inducing signal. As stress inducers we have used antimycobacterial drugs that target the biogenesis of the mycobacterial cell envelope, as these have previously been shown to specifically induce the major cell wall stress operon *iniBAC*. We have identified a multi-domain regulator that is essential for the induction of this operon to transduce cell envelope stress and named this IniR. Importantly, we were also able to show that cell envelope stress signaling was induced by free trehalose. Trehalose is a central unit in many mycobacterial lipids and mycobacteria have a dedicated trehalose salvage pathway that is used when lipids are degraded and recycled. We hypothesize that lipid turnover and concomitant release of free trehalose in the cell envelope is a signal for cell envelope stress in mycobacteria.

## Introduction

*Mycobacterium tuberculosis*, the causative agent of tuberculosis disease (TB), is currently the most deadly infectious agent, causing over 1.8 million deaths annually [1]. Although TB can be cured with a six-month treatment regimen of different antibiotics and chemotherapeutics, this disease is still a huge public health burden in large parts of the world [1]. One of the major issues in combating TB is the emergence of multi-drug (MDR) and extensively-drug resistant (XDR) strains. To combat TB caused by these new strains novel anti-tubercular agents are urgently needed. A prominent drug target is the mycobacterial cell wall, a non-canonical and lipid-rich structure [2]. One of the unique elements of the mycobacterial cell wall is the so-called arabinogalactan layer that is covalently linked to the peptidoglycan. This layer is composed of a chain of galactofuranose residues and side chains of (branched) arabinofuranose units. The terminal arabinofuranose residues of these side chains can be substituted with long (C60-C90) carbon chain fatty acids, commonly known as mycolic acids. These mycolic acids form the inner layer of another unique element, the mycobacterial outer membrane. Mycolic acids can also be linked to trehalose, resulting in trehalose mono- (TMM) and trehalose dimycolates (TDM). These glycolipids are hypothesized to form the outer layer of the outer membrane. The mycobacterial outer membrane also contains a variety of other (glyco)lipids, many of which contain a trehalose unit [3].

The success of targeting mycobacterial cell envelope biogenesis with antitubercular drugs is best illustrated by first-line antibiotics ethambutol (EMB) and isoniazid (INH) and recently discovered antibiotics like benzothiazinone (BTZ). Both EMB and BTZ disrupt the formation of the arabinogalactan layer; EMB targets arabinosyltransferases EmbA, EmbB and possibly EmbC, which effectively abolishes the biosynthesis of arabinogalactan and lipoarabinomannan [4,5]. BTZ, in turn, inhibits arabinan biosynthesis by binding to the decaprenylphosphoryl-β-D-ribofuranose-2'-epimerase enzyme DprE1 [6,7]. INH enters the mycobacterial cell as a pro-drug and, after intracellular activation, binds and inhibits InhA, an essential NADH-dependent enyol-ACP reductase that is required for mycolic acid biosynthesis. Inhibition by INH causes severe cell wall deformations, ultimately leading to bacterial cell death [8,9].

Mycobacterial cell wall composition and biosynthesis have been studied extensively. However, the induction and function of mycobacterial cell envelope stress responses are largely unexplored. Mapping bacterial stress pathways is important, because key components that are essential for dealing with stress, for instance as a result of antibiotic treatment with cell-wall targeting antibiotics or chemotherapeutics can be identified. This knowledge can subsequently shed light on possible resistance routes that subvert the current treatment regimen. Moreover, pinpointing these stress-associated networks may lead to new targets for anti-TB therapy or lead to the discovery of antimycobacterial agents that can synergize with currently used antibiotics. Also, identification of important antibiotic-associated regulatory networks can lead to the discovery of sensitizing agents, as shown previously by work from Peterson et al. [10]. A recent example of a sensitizing agent is the recently identified ethR2-inactivating agent, which leads to reversion of ethionamide resistance [11]. Studying stress responses can also provide fundamental insights into the bacterial heterogeneity observed in patients receiving treatment.

To fill the gap in our knowledge on mycobacterial cell envelope stress responses, we study *iniBAC* operon induction. This operon is the most abundantly induced gene cluster upon incubation with sub-lethal concentrations of EMB and INH [12]. We and others have previously shown that this operon is specifically induced as a result of cell wall stress [12,13]. For our studies we used the causative agent of fish tuberculosis, *Mycobacterium marinum*, and confirmed our findings for *M*. *tuberculosis*. A previous transposon mutagenesis study performed by our group revealed that the *iniBAC* operon was strongly induced by mutations in vitamin B12 biosynthesis genes or in genes encoding the vitamin B12-dependent enzyme methylmalonyl-CoA mutase (MutAB) [13]. Because MutAB is a crucial component of the proprionate-degradation pathway, we hypothesized that *iniBAC* upregulation may be linked to coping with otherwise toxic levels of metabolic intermediates or that it may influence the production of (trehalose-containing) branched chain fatty acids [14]. However, the genes directly involved in regulation of the *iniBAC* operon remained unknown. Other groups have speculated on possible regulators of the *iniBAC* operon, but none of them can explain the high, specific upregulation of this single operon during antibiotic stress conditions [15,16,17]. In this study, we aimed to identify the regulator of the *iniBAC* operon. We used a transposon mutant screen to identify the activator that is essential and specific for *iniBAC* regulation.

## Results

### Identification of mutants unable to induce the *iniBAC* operon

Previously, we have shown that mutants affected in vitamin B12-biosynthesis and the vitamin B12-dependent enzyme MutAB already displayed a high degree of *iniBAC* induction in the absence of any antibiotic [13]. These transposon mutants with upregulated *iniBAC* expression did not show an apparent phenotype, *e*.*g*. we did not observe an altered susceptibility to first-line antibiotics, nor a growth defect in culture [13]. As an indicator for *iniBAC* induction we used a construct containing the *iniBAC* promoter cloned in front of a promoterless gene encoding the fluorescent protein mEos3.1. To identify the *iniBAC* regulator, we used one of the previously described vitamin B12 biosynthesis mutants, *cobC*::*tn*, carrying an integrative variant of the *iniBAC* reporter plasmid and performed a second round of transposon mutagenesis [13]. This time, mutant colonies that resulted from transposon mutagenesis were selected for the absence of fluorescence on 7H10 plates, as assessed manually, by fluorescence microscopy. Next, these mutants were streaked on fresh 7H10 plates and compared to a WT *M*. *marinum* containing the reporter, as a negative control. We termed these non-fluorescent mutants ‘on/off mutants’. In total, 27 transposon mutants that repressed *iniBAC* induction were characterized, of which 2 could be retraced to mutations in the *mEos3*.*1* gene of the reporter construct. The remaining 25 on/off mutants were all mapped to specific transposon insertion locations within the *M*. *marinum* genome (Table 1). With 5 independent transposon insertions, the *mce1* operon was most prominently present in our mutant list. Although this operon is not directly involved in gene regulation, we decided to characterize these mutants in more detail because they could reveal clues on the signal transduction pathway that is involved in *iniBAC* induction.

**Table 1:** List of *M*. *marinum* mutants that showed repression of *iniBAC* constitutive induction in the *cobC*::*tn* strain.

Gene ID	Gene product	H37Rv orthologue	Gene length (bp)	Unique mutants (TA position from 5’ of gene)
*MMAR_0076*	DnaB	*Rv0058*	1383	1196
*MMAR_0411*	YrbE1B	*Rv0168*	870	17
*MMAR_0413*	Mce1B	*Rv0170*	1041	791, 800
*MMAR_0414*	Mce1C	*Rv0171*	1566	11
*MMAR_0415*	Mce1D	*Rv0172*	1608	283
*MMAR_0511*	SdhA_1	*Rv0248c*	1929	1885
*MMAR_0612*	IniR	*Rv0339c*	2454	290, 392, 1780
*MMAR_0831*	CmaA2	*Rv0503c*	912	124
*MMAR_1706*	LppZ	*Rv3006*	1122	397
*MMAR_1803*	LppW	*Rv2905*	960	628
*MMAR_2219*	MMAR_2219	*Rv1410c*	1554	1157
*MMAR_2286*	MoxR1	*Rv1479*	1116	22
*MMAR_2628*	MMAR_2628	-	999	223
*MMAR_3294*	GlnA2	*Rv2222c*	1341	1093, 1312
*MMAR_3334*	AceE	*Rv2241*	2790	187, 2569
*MMAR_4169*	PknH_1	*Rv1266c*	1854	46
*MMAR_4184*	Cyp130A4	*Rv1256c*	1242	1006
*MMAR_4717*	Cyp188A3	-	1365	331
*MMAR_4806*	CrtI	-	1530	1332
*MMAR_5423*	MMAR_5423	-	1206	143

The H37Rv orthologue, if present, the gene length and the number of unique mutants per gene are indicated. Unique mutants are annotated by the position of the transposon insertion from the 5’ of the gene.

### Selected *mce1* operon mutants show altered levels of free and bound mycolic acids

**Fig 1:**
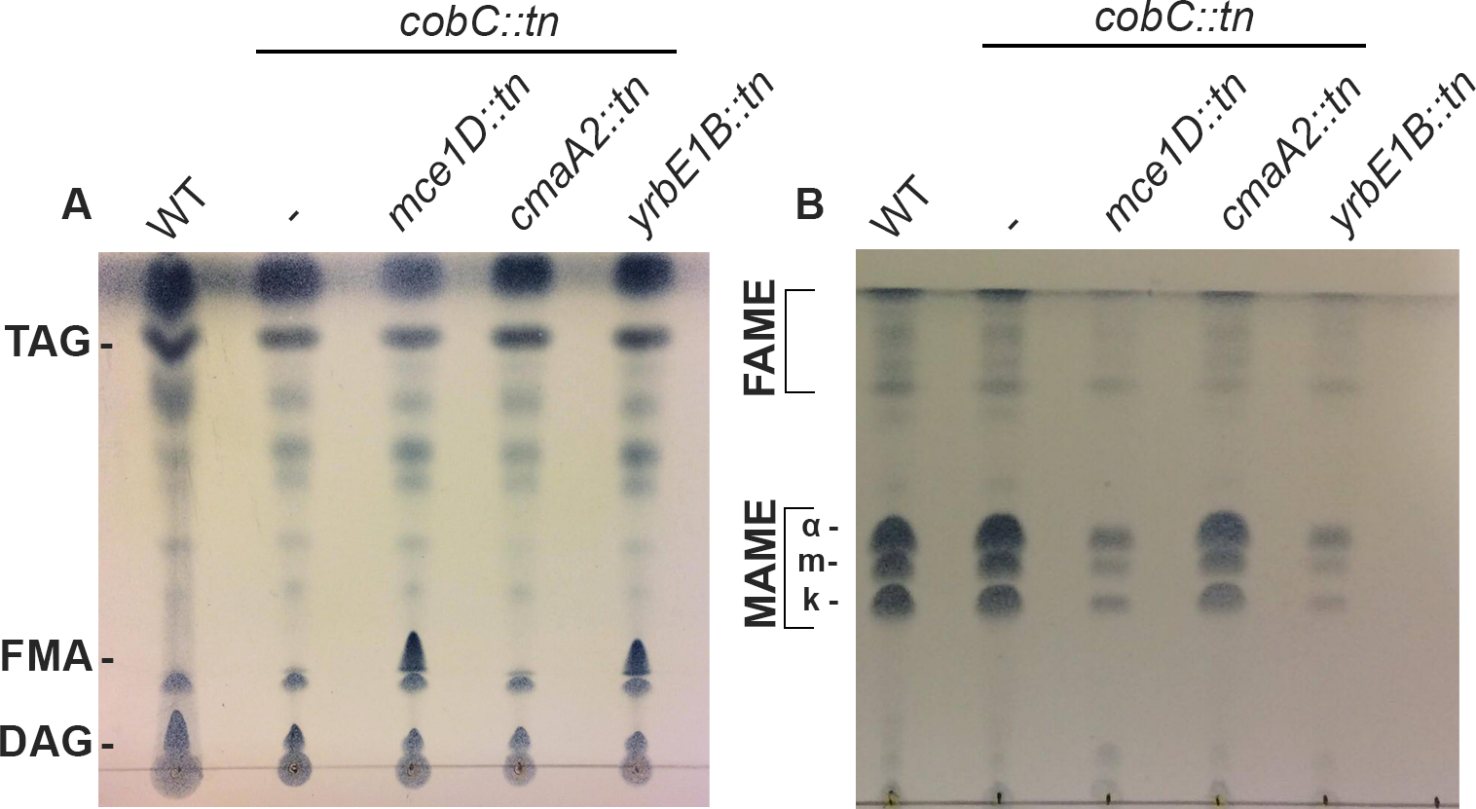
Lipid profiles of *mce1*::*tn* mutants show an increase in free mycolic acid and a decrease in bound mycolic acids. TLC analysis of lipid extracts from *M*. *marinum* WT strain, *the cobC*::*tn* strain used for the transposon mutagenesis and three on/off mutants that showed decreased *iniBAC* induction, *i*.*e*. *mce1D*::*tn*, *yrbE1B*::*tn* and *cmaA2*::*tn*. TLC loading was normalized by OD and further adjusted according to pellet weight to ensure equal amounts of lipid were spotted. (A) TLC of polar lipids, triacylglycerols (TAG), free mycolic acids (FMAs) and diacylglycerols (DAG) are distinguished. A clear increase in FMAs can be seen for both *mce1* operon mutants. (B) TLC analysis of the different types of bound mycolic acids (MAMEs and FAMEs) where for MAMEs alpha (α), keto (k) and methoxy (m) species are indicated. There is a clear decrease in bound mycolic acids for *mce1D*::*tn* and *yrbE1B*::*tn*.

It has been reported that disruption of the *mce1* operon in *M*. *tuberculosis* results in the intracellular accumulation free mycolic acids (FMAs) [18], indicating that Mce1 is involved in the reuptake of FMAs from the cell envelope [18,19]. To identify whether *M*. *marinum mce1* transposon mutants also show an aberrant lipid profile, thin layer chromatography (TLC) was performed to measure FMA levels. In line with these previous findings, disruption of both *mce1D* and *yrb1EB* of the *mce1* operon resulted in a substantial increase in FMAs, as compared to WT *M*. *marinum* and the parent *cobC*::*tn* strain (Fig 1A) [18]. As a control, a double mutant affected in the cyclopropanation of mycolic acids, *cmaA2*::*tn* was included. This mutant seemed to be unaffected in the level of FMAs. In addition to FMAs, the amount of bound mycolic acids were also analyzed. Interestingly, both *mce1* mutants displayed a large decrease in the amount of bound mycolic acids, as compared to the parent strain (Fig 1B). We hypothesized that a decrease in bound mycolic acids might influence membrane integrity and thus cell envelope stress. To test this hypothesis, we performed an MIC assay with a selection of first and second line antibiotics (ciprofloxacin, ethambutol, isoniazid and rifampicin). In the experiment, a WT *M*. *marinum* M^USA^, the *cobC*::*tn* parent strain and the double mutants affected in *yrbE1B* and *mce1D* were included. However, no major differences in the MICs between the *mce1D*, *yrbE1B* transposon mutants and the respective controls were observed (S1 Table). To assess cell wall permeability with a different method, we also performed an ethidium bromide uptake assay with a WT *M*. *marinum*, the *cobC*::*tn* parent mutant and the *mce1D*::*tn* + *cobC*::*tn* double mutant. We used a WT *M*. *marinum* that expresses *M*. *smegmatis* porin MspA as a positive control, because it has previously been shown to have an increased outer membrane permeability [20]. As expected, we observed a high increase in permeability for our positive control (S1 Fig). However, both *cobC*::*tn* and the *mce1*::*tn* on/off mutant showed highly similar rates of ethidium bromide uptake when compared with our WT control. Therefore, we conclude that although *mce1* mutants seem to be affected in the mycolic acid salvage pathway and show reduced *iniBAC* induction, this mutation does not seem to affect cell envelope permeability.

### *iniBAC* induction by EMB and INH is abrogated in *iniR* mutants

**Fig 2:**
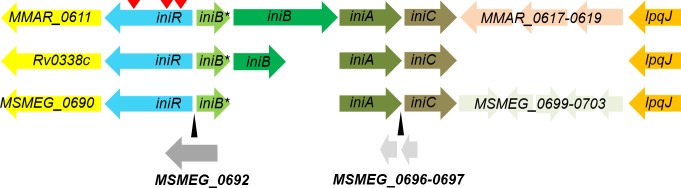
An overview of the *iniR-*containing regions in mycobacteria. The species containing *iniR* (blue) are *M*. *marinum* (top), *M*. *tuberculosis* (middle) and *M*. *smegmatis* (lower). Conserved genes are indicated by identical colors. Insertions of genes in the genome of *M*. *smegmatis* are indicated with the black arrows. Distances are proportional to actual size. Red triangles indicate the approximate positions of the 3 independent transposon insertions in *iniR* that affect *iniBAC* induction in *M*. *marinum*.

**Fig 3:**
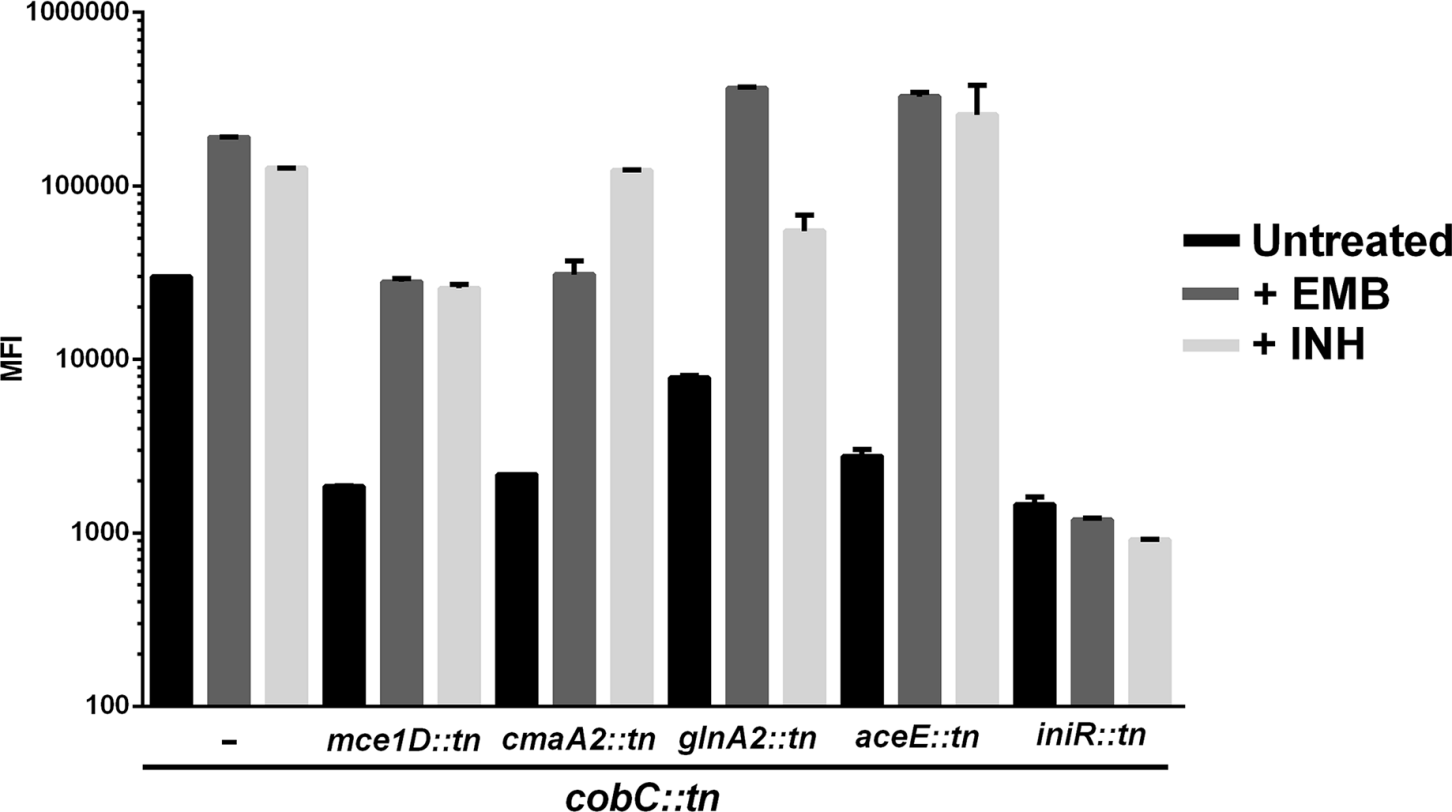
The *iniR*::*tn* mutants are unable to induce *iniBAC* upon addition of EMB or INH. Five on/off mutants are displayed in this graph to visualize induction of *iniBAC* upon treatment with EMB or INH as analyzed by flow cytometry. The first 3 columns show the *cobC*::*tn* parent strain, which is clearly induced by default (black bar), but further inducible by EMB (1 μg/ml, dark grey bar) or INH (10 μg/ml, light grey bar). All the other mutants are derived from the *cobC*::*tn* parent strain and the second mutated gene is indicated below the bars. The mutant affected in *MMAR_0612* (*iniR*_*Mm*_) is the only one in the panel shown here that shows no upregulation of *iniBAC*. Measurements were performed in triplicate and the error bars indicate the standard deviation.

The second most abundant set of mutations in our transposon screen were three unique insertions in one gene, *MMAR_0612*. The gene product of *MMAR_0612* has a predicted DNA-binding domain, suggestive of a role as transcriptional regulator (as determined by Phyre 2 [21]) and is located upstream of the *iniBAC* operon (Fig 2). Together, these characteristics make this gene a major candidate to code for the *iniBAC* regulator. To examine this, we tested whether the reporter construct present within the *MMAR_0612* on/off mutants could still be induced by EMB (1 μg/ml) or INH (10 μg/ml) in culture. As a control, we included a selection of other on/off mutants (*e*.*g*. we display one *mce1* transposon mutant and one *iniR*::*tn* mutant). The on/off mutants were cultured with or without 1x MIC EMB (1 μg/ml) or INH (10 μg/ml), both concentrations that were previously established to cause a strong induction of our reporter plasmid [13]. The response was quantified by measuring fluorescence induction with a flow cytometer (a representative selection is shown in Fig 3). Out of the 25 mutants identified we found only three mutants that were unresponsive and showed no induction of *iniBAC* upon antibiotic challenge. These mutants were all affected in *MMAR_0612*, indicating that this is indeed the *iniBAC* regulator. Consequently, we propose to name the *MMAR_0612* gene product IniR for ***ini****BAC* **R**egulator. Next, a MIC assay was performed with first- and second-line antimycobacterial compounds ciprofloxacin, ethambutol, isoniazid and rifampicin on one of the *iniR*::*tn* mutants to address whether disruption of this gene altered antibiotic susceptibility. Comparing the MIC values of *iniR*::*tn* to the *cobC*::*tn* parent strain and an *sdhA1*::*tn* ‘on/off mutant’ revealed that there are no major differences in antibiotic sensitivity (S1 Table). This finding is in line with previous evidence that constitutive upregulation of the *iniBAC* operon did not change antibiotic susceptibility [13].

### Complementation of *iniR* restores *iniBAC* induction

**Fig 4:**
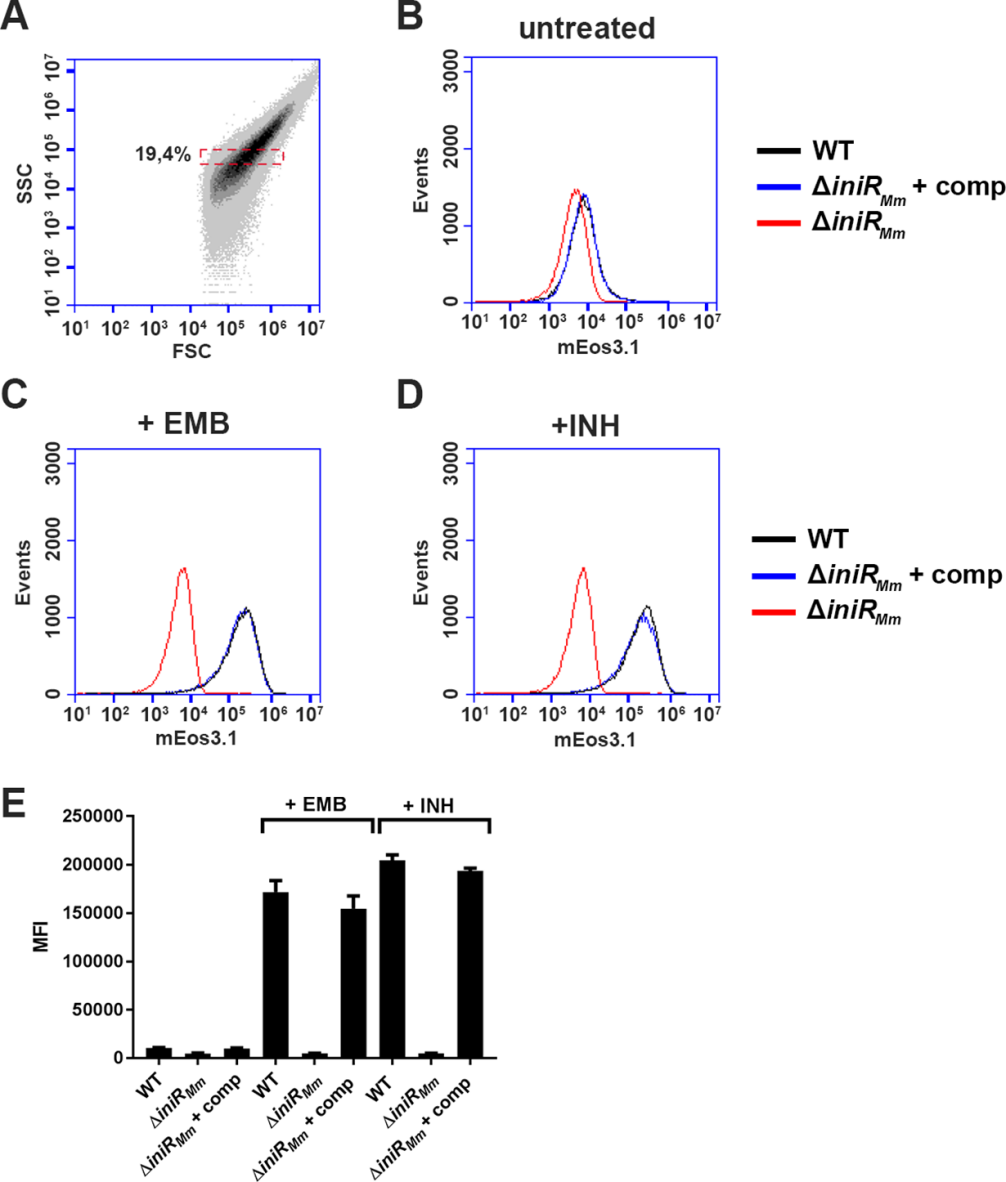
A Δ*iniR*_*Mm*_ mutant is unable to induce *iniBAC* upon addition of EMB or INH. (A) Gating strategy for this experiment. The bacterial population in the red box was selected to measure a population that is roughly equal in size and granularity (measured by forward scatter, FSC and side scatter, SSC). This gated population was used for all samples and a total of 30,000 cells were analyzed per sample (B) Histograms of mEos3.1 fluorescence intensity (in arbitrary units, on the X-axis) of untreated WT *M*. *marinum* (black line), an isogenic *iniR*_*Mm*_ knockout mutant and the *iniR*_*Mm*_ knockout mutant transformed with an integrative plasmid containing a copy of *iniR*_*Mm*_ with its native promoter are shown for day 3. (C) Fluorescence induction by EMB (1 μg/ml) is lost in the *iniR*_*Mm*_ mutant, but restored in the complemented strain to near-WT levels. (D) Fluorescence induction by INH (10 μg/ml) is lost in the *iniR*_*Mm*_ mutant, but also restored in the complemented strain to near-WT levels. The histograms are from one representative sample of a biological quadruplicate (E) Quantification of the fluorescence induction (in mean fluorescence intensity, MFI) measured on day 3. Measurements were performed in quadruplicate. Error bars indicate s.d. values.

To confirm our previous results, as well as to exclude possible side-effects of the *cobC*::*tn* mutant background, we generated a targeted knockout of *MMAR_0612* in *M*. *marinum* (from here onwards termed *iniR*_*Mm*_). A complementation vector that contained the putative native *iniR*_*Mm*_ promoter and *iniR*_*Mm*_ was also constructed and introduced into the Δ*iniR*_*Mm*_ strain. Subsequently, the *iniBAC* reporter plasmid was introduced into the Δ*iniR*_*Mm*_ and the complemented Δ*iniR*_*Mm*_ strain and cultures of the resulting strain were treated with either 1x MIC EMB or 1x MIC INH and compared to a control culture without antibiotics. On day 3, cultures were washed and bacterial cells were analyzed for fluorescence induction by flow cytometry. The gating strategy for this experiment can be found in Fig 4A. As shown in Fig 4B–4D, the Δ*iniR*_*Mm*_ mutant showed no induction upon treatment with EMB or INH, confirming previous findings with the *iniR*_*Mm*_::*tn* mutants in the *cobC*::*tn* background. Upon complementation *iniBAC* induction levels were restored back to near wild-type levels. In addition, we also observed a mild (2-fold) repression of *iniBAC* in the knockout mutant grown without antibiotics, indicating that IniR_Mm_ is also required for basal production levels of the *iniBAC* operon (Fig 4E)

### *iniR* is an unconventional regulatory element that is conserved among mycobacterial species

Bioinformatic analysis of the locus of *iniR* and *iniBAC* reveals that the synteny is conserved in several mycobacterial species, including *M*. *tuberculosis*, *M*. *bovis* and *M*. *smegmatis* (Fig 2). In all these species, the gene encoding the IniR regulator is separated from the *iniBAC* operon by a small gene designated iniB*, because this gene shows weak homology to the 5’ end of *iniB* (32% identity over a length of 50 amino acids for the *M*. *marinum* proteins). The major differences in the *iniBAC* locus are caused by the presence and the size of the *iniB* gene; *M*. *smegmatis* lacks an *iniB* gene, whereas the *iniB* gene of *M*. *marinum* is, with 3000 bp, more than double the size of *M*. *tuberculosis iniB*. Moreover, *M*. *smegmatis* contains a *dnaK*-like gene directly downstream of *iniR*.

**Fig 5:**
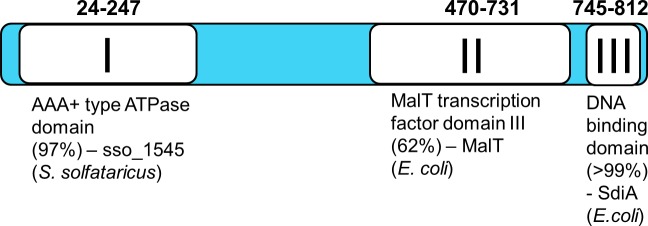
Overview of the three domains that can be found in *iniR*. Noted domains are the most significant hits using Phyre2 structure prediction [21]. The number between brackets indicates the confidence with which the Phyre2 prediction was made. A combination of NCBI BLAST and the Phyre2 server was used to obtain domain predictions. Domain I (amino acid 24 to 247) is an AAA+ ATPase domain sharing 15% amino acid identity with sso_1545 in *S*. *solfataricus*. Domain II (470–731) is 16% identical to domain III of MalT in *E*. *coli* and the C-terminal domain (745–812) contains a DNA binding domain sharing 20% identity to *E*. *coli* SdiA. Displayed domains are proportional to actual domain size.

Bioinformatic analysis (NCBI BLAST and Phyre2 [21]) of the 818 amino acids-long IniR of *M*. *marinum* revealed three distinct domains (Fig 5). The first domain is an AAA+ ATPase domain, located at the N-terminal side of the protein. This domain is predicted to be structurally highly similar to sso_1545 from *Sulfolobus solfataricus*, an archaeal ATPase [22]. A second domain (from AA 470 to AA 731), shows structural similarity (Phyre2) to domain III of MalT, a transcription factor found in *E*. *coli*. This domain is responsible for binding maltotriose, which subsequently triggers multimerization of the transcription factor MalT [23]. In *E*. *coli* MalT is coupled to the maltose uptake system and, upon binding maltose, positively regulates transcription of the genes encoding the maltose transporter (*mal* operon) [24]. Lastly, the C-terminal portion of IniR contains a putative DNA-binding domain, showing the highest similarity to SdiA, a LuxR-like regulator in *E*. *coli*. In conclusion, the bioinformatics analysis indicates that IniR is a multidomain regulator that is responsive to external effectors.

### IniR can bind the *iniBAC* promoter and induce transcription in a modified reporter assay

**Fig 6:**
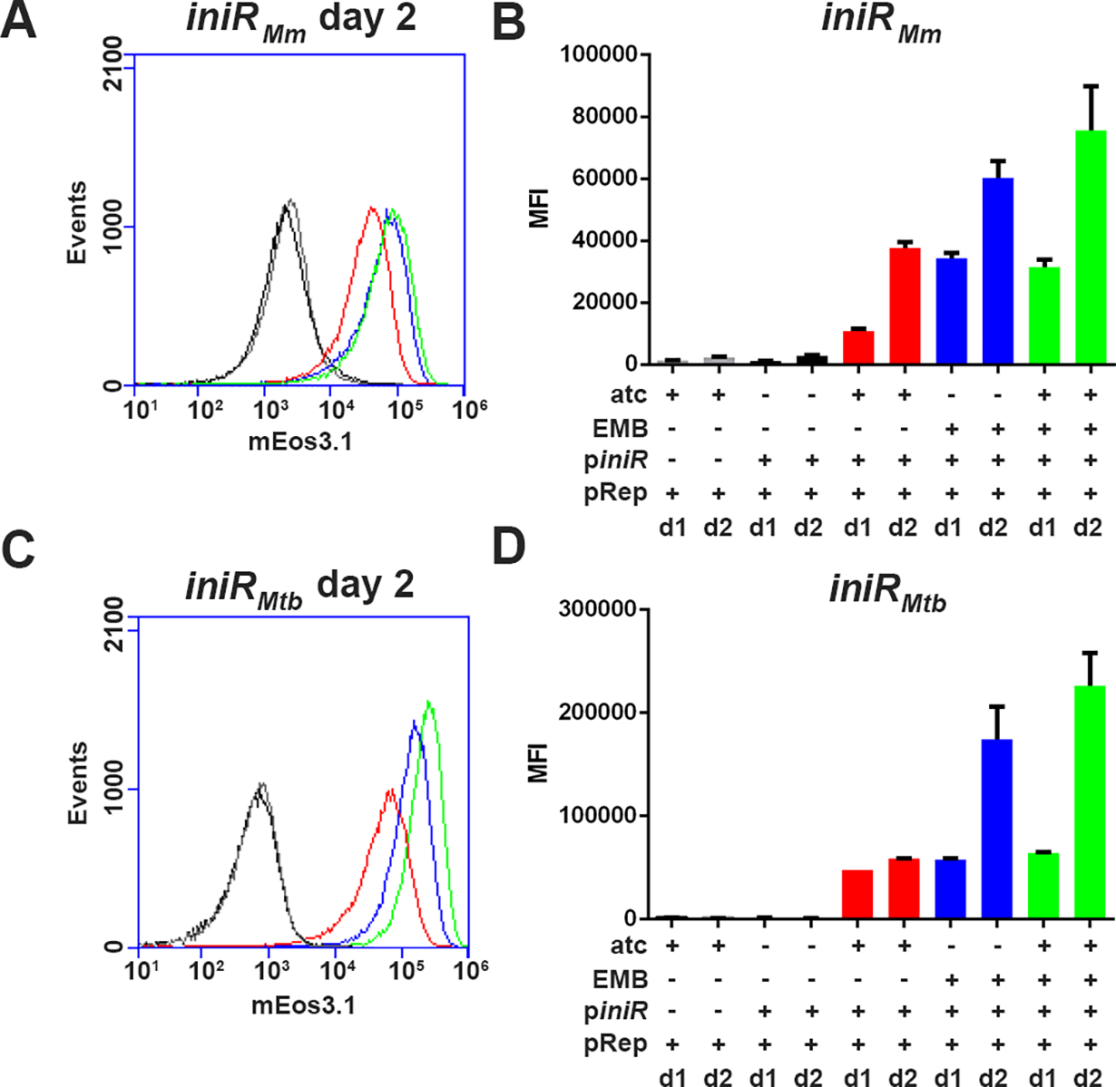
overexpression of *iniR* induces *iniBAC* transcription. Overexpression of either *M*. *marinum* (IniR_Mm_) or *M*. *tuberculosis* (IniR_Mtb_) *iniR* in *M*. *smegmatis* results in antibiotic-independent induction of the *iniBAC* promoter on the reporter plasmid (pRep). *M*. *smegmatis* containing the *iniBAC* reporter plasmid and the ATc inducible *iniR*_*Mm*_ (*M*. *marinum* gene *MMAR_0612*) (A) or *iniR*_*Mtb*_ (*M*. *tuberculosis* gene *Rv0339c*) (C). The presence of the *iniR-*containing plasmid is indicated with p*iniR*. We compared presence of both plasmids and ATc (in red) to culturing without ATc (in black). We also checked influence of the presence (+) of EMB alone (in blue) and in the presence of both ATc and EMB (in green). *M*. *smegmatis* containing only the reporter plasmid and cultured with ATc was used as a control (in grey). (B) and (D) are quantifications of (A) and (C), including day 1 data. The presence (+) or absence (-) of plasmids or EMB/ATc is indicated. The experiment was performed in triplicate. The error bars indicate the s.d. values. The MFI is the mean fluorescence intensity of 30.000 gated cells (The gating strategy shown in Fig 4A was used). Fluorescence intensity (a.u.) of mEos3.1 is depicted on the X-axis for panels (A) and (C).

**Fig 7:**
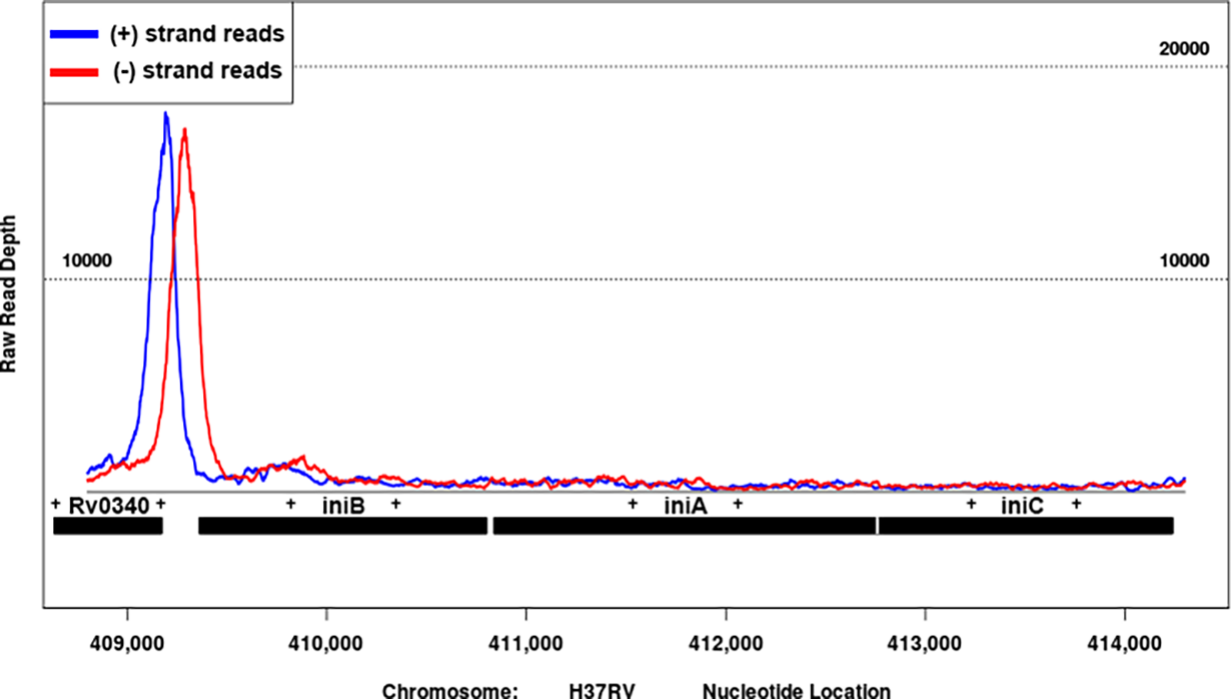
ChIP sequencing shows a clear binding peak for IniR_Mtb_ upstream of the *iniBAC* operon. Chromatin immunoprecipitation (ChIP) sequencing was performed to identify binding events of IniR_Mtb_-FLAG_._ To do so *M*. *tuberculosis* containing a vector encoding the FLAG-tagged IniR_Mtb_ was induced with ATc and crosslinked after induction. After lysis, chromatin was sonicated to small fragments and immuno-precipitated with anti-FLAG antibody. And purified and sequenced. A clear binding peak of IniR_Mtb_ upstream of the *iniBAC* operon could be identified. The blue line indicates the (+) strand reads and the red line indicates the (-) strand reads. The number on the X-axis represent the nucleotide position in the H37Rv genome.

Based on our results and the bioinformatic analysis, we hypothesized that IniR is a direct activator of *iniBAC*. To test this, we created an anhydrotetracyclin-inducible (ATc) expression vector that encodes a FLAG-tagged *M*. *marinum* IniR (IniR_Mm_) and introduced the construct in an *M*. *smegmatis* strain that also contained the *iniBAC* reporter plasmid. The resulting strain was grown in presence or absence of 10 ng/ml ATc and fluorescence induction was assessed with flow cytometry. At 2 days after induction a strong, ATc-dependent, mEos3.1-signal was observed and this induction was specific for the presence of the vector containing *iniR*_Mm_ (named p*iniR* in Fig 6A). The induction of fluorescence by ATc compared very well to that of the known inducer EMB. Moreover, addition of EMB combined with ATc boosted induction to approximately 1.5 times the observed induction with ATc alone (Fig 6B). In order to investigate whether *Rv0339c* (*iniR*_Mtb_) of *M*. *tuberculosis* is indeed the functional orthologue, we used an ATc-inducible vector encoding iniR_Mtb_-FLAG and repeated the same experiment. With the FLAG-tagged IniR_Mtb_ we observed similar induction pattern after addition of ATc (Fig 6C and 6D), indicating that IniR_Mtb_ is indeed the functional orthologue. To confirm that IniR_Mtb_ binds to the promoter region of the *iniBAC* operon to induce transcription, we also performed a chromatin immuno-precipitation (ChIP) sequencing experiment. Briefly, *M*. *tuberculosis* containing the ATc inducible vector encoding IniR_Mtb-_FLAG was cultured in the presence of ATc. Cultures were crosslinked and bacteria lysed. Subsequently DNA-bound iniR_Mtb_-FLAG was immuno-precipitated with an anti-FLAG antibody. DNA fragments were de-crosslinked, purified and sequencing was used to determine the binding DNA regions. The resulting reads were mapped onto the H37Rv genome and showed a very strong binding peak of IniR_Mtb_ directly in front of the *iniBAC* operon (Fig 7). Together these experiments show that expression of IniR is sufficient to induce the *iniBAC* operon. Moreover, IniR_Mtb_ can very clearly bind directly upstream of the *iniBAC* operon, indicating that IniR is the *iniBAC* activator protein in both *M*. *marinum* and *M*. *tuberculosis*.

### IniR only induces the *iniBAC* operon

**Fig 8:**
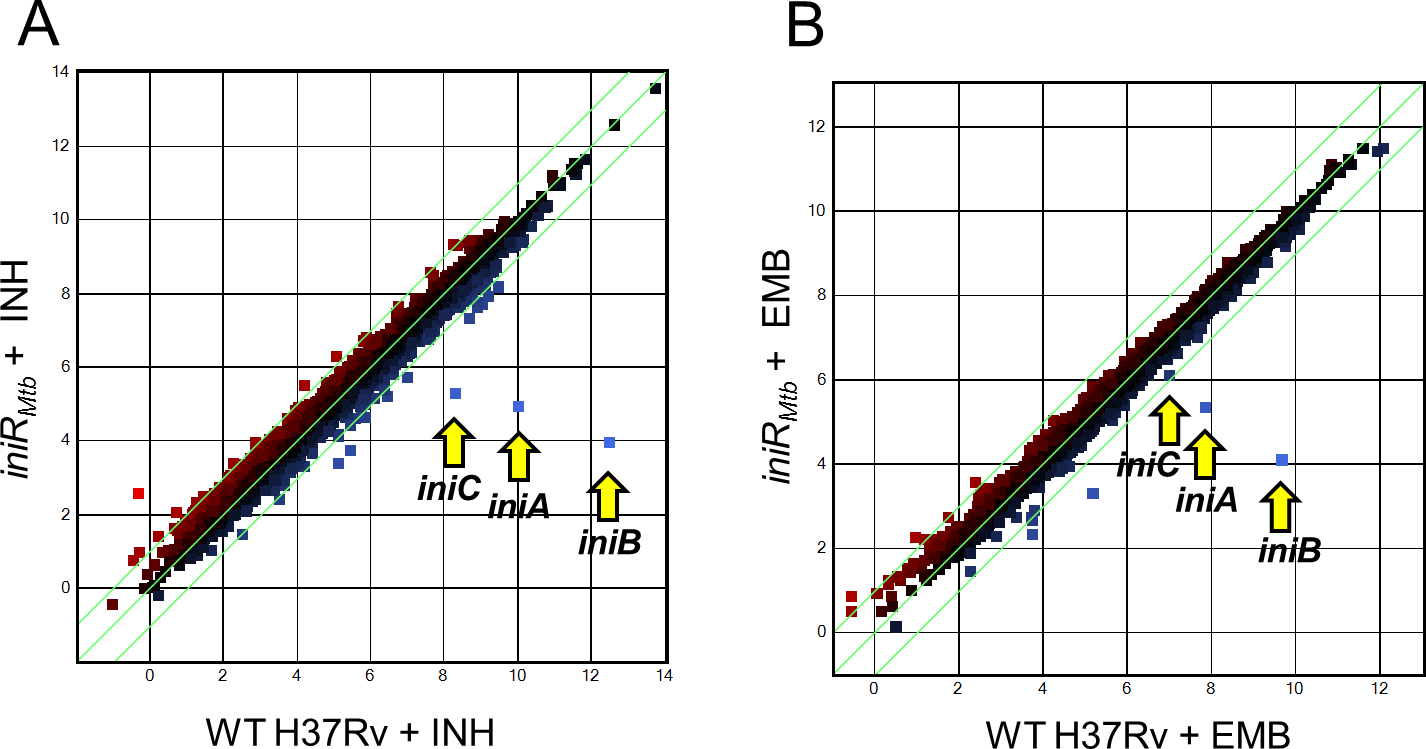
RNA sequencing data of *M*. *tuberculosis* indicates that only the *iniBAC* operon is regulated through IniR. (A) The plots contain gene expression profiles in log2 fold change reads per kilobase million (RPKM) of Δ*iniR*_*Mtb*_ + INH compared to the control strain, *i*.*e*. *M*. *tuberculosis* H37Rv WT + INH. (B) Plotted gene expression profile in log2 fold change RPKM of Δ*iniR*_*Mtb*_ + EMB compared to H37Rv WT + EMB. The *iniBAC* genes are indicated with yellow arrows. The experiment was performed with a biological triplicate.

To determine the IniR regulon we used a previously generated *M*. *tuberculosis* H37Rv *iniR*_*Mtb*_ mutant. Using this strain, we performed mRNA sequencing using different growth conditions, comparing the RNA profiles of untreated, EMB- or INH-treated cultures of WT H37Rv to those of the *iniR*_*Mtb*_ knockout strain. Consistent with prior observations, the *iniBAC* operon was highly induced in WT H37Rv *M*. *tuberculosis* after treatment with either ethambutol or isoniazid. Knocking out *iniR*_*Mtb*_ completely abrogated the induction with both antibiotics (Fig 8A and 8B), similar to our results for *iniR*_*Mm*_ (Fig 4). When comparing the expression in WT versus the *iniR*_*Mtb*_ knockout mutant, *iniBAC* levels were reduced ~6 fold on average in untreated conditions (S2 Table), a similar repression as seen for *iniR*_*Mm*_ disruption. Values in S2 Table are fold change in RNA levels for a triplicate of samples comparing WT H37Rv to Δ*iniR*_*Mtb*_. Treatment with EMB or INH does not increase transcription of the *iniR*_*Mtb*_ activator itself, indicating that this element is not regulating its own expression by upregulation of *iniR*_*Mtb*_ transcripts. An important observation is that only the expression of the *iniBAC* operon was significantly altered when *iniR*_*Mtb*_ was deleted, which implies that IniR is a highly specific and dedicated activator, only regulating this single operon.

### Transcription of *iniBAC* can be directly activated by the addition of trehalose

**Fig 9:**
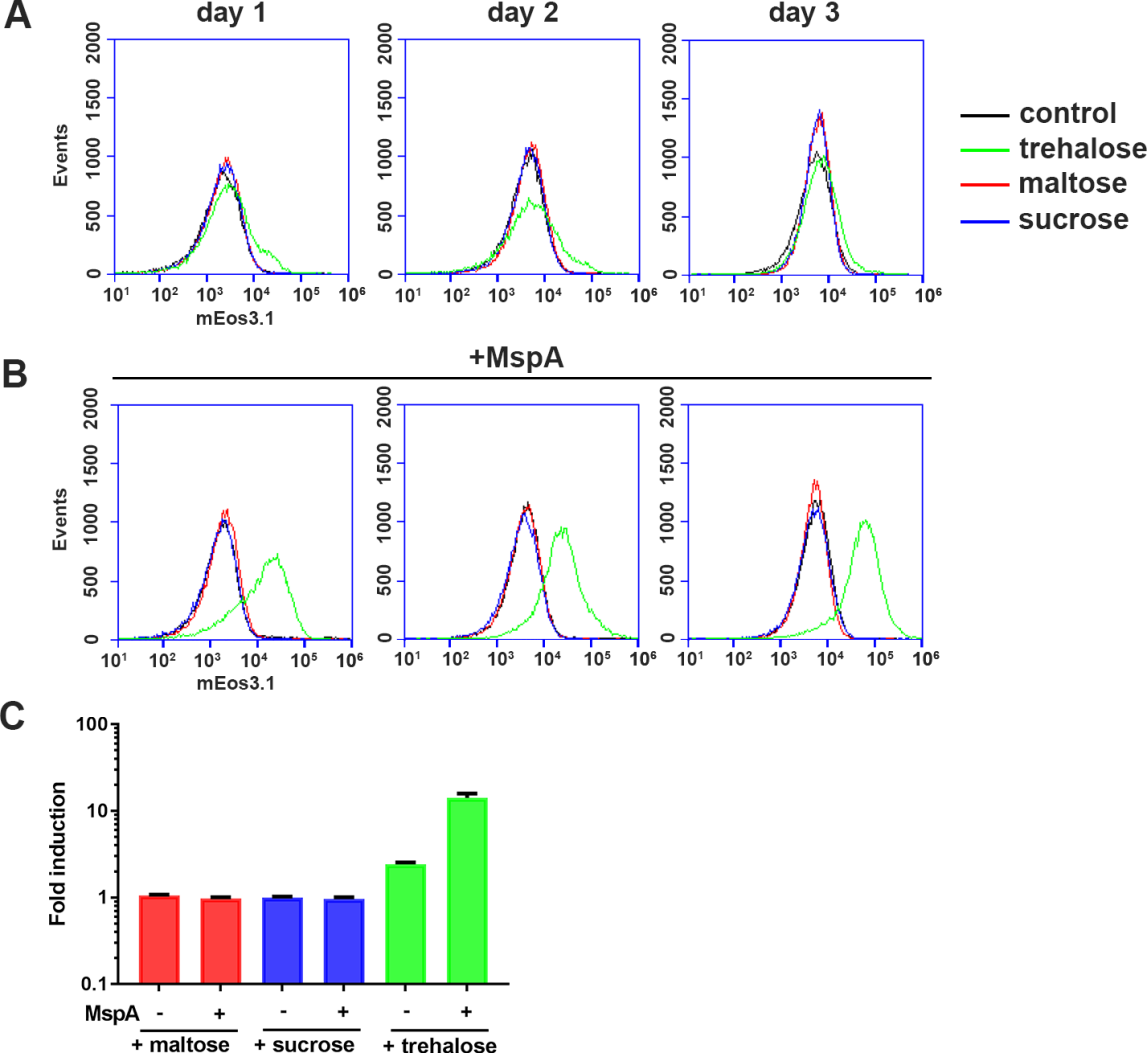
Addition of trehalose is sufficient to induce *iniBAC* induction in *M*. *marinum*. The effect of additional trehalose is especially prominent when the outer membrane is more permeabilized by the expression of the MspA porin of *M*. *smegmatis*. (A) Flow cytometry histograms of induction levels of a WT *M*. *marinum* containing the *iniBAC* reporter upon treatment with either 1% trehalose (green), 1% maltose (red), 1% sucrose (blue) or no disachharide (black line). In the graph plots are shown for day 1, 2 and 3 after addition. The graphs are representative images of one experiment performed in triplicate. (B) Same as in (A) but this experiment the strains contained an additional plasmid encoding porin MspA. Fluorescence intensity of mEos3.1 (in arbitrary units) is plotted on the X-axis for all six histograms (C) A quantification of the fold induction on day 3 of three independent experiments. The fold induction was calculated by dividing the MFI of the treated sample to the MFI of the corresponding untreated control. The presence of MspA is indicated with + and -, as well as the different sugars tested; maltose (red), sucrose (blue) and trehalose (green). s.d. values are indicated by the error bars. Cultures were grown in 7H9 containing 0.2% glycerol and 0.05% Tween-80.

Because IniR has structural homology to the MalT domain III that is responsible for maltotriose binding, we hypothesized that the presence or uptake of (di)saccharides may be triggering IniR multimerization and subsequent activation of *iniBAC*. To test this hypothesis, WT *M*. *marinum* containing an integrated *iniBAC* reporter plasmid was grown in the presence of maltose, sucrose or trehalose. The introduction of the *iniBAC* reporter on the chromosome was chosen to simulate a more natural expression level of *iniBAC*, even though an integrated variant reduces the dynamic range of the induction levels than can be observed. After exposure to maltose, sucrose and trehalose, fluorescence induction was measured by flow cytometry after one, two and three days of incubation. Both 1% maltose and 1% sucrose did not induce *iniBAC*. However, addition of 1% trehalose caused a small but reproducible 2.5 fold induction (Fig 9A and 9C), indicating that trehalose can induce *iniBAC* expression. Trehalose plays an important role in mycobacterial cell envelope biogenesis. A well-known example is the release of trehalose when TDMs are produced from two TMM molecules. To determine whether the levels of free mycolic acids were influencing *iniBAC* induction by trehalose, we assessed an *cobC*::*tn* + *mce1D*::*tn* mutant for induction of our fluorescent *iniBAC* reporter. However, the *mce1D*::*tn* double mutant showed similar induction levels as the control transposon mutants (S2 Fig), indicating that the sensing and signaling of *iniBAC* induction is separate from the reimport of the trehalose-containing lipid TMM.

### *iniBAC* induction by trehalose is dependent on cell wall permeability

We hypothesized that outer membrane permeability could be a major factor in the limited induction levels observed upon addition of 1% trehalose. Therefore, a plasmid containing the gene coding for the *M*. *smegmatis* porin MspA, was introduced [20]. Introduction of MspA has previously been shown to increase the outer membrane permeability in slow-growing mycobacteria, which was also confirmed by our EtBr uptake experiment (S1 Fig) [20]. The resulting indicator strain with increased outer membrane permeability showed a significant increase in the ability to induce *iniBAC* in response to 1% trehalose (Fig 9B and 9C); a 12-fold induction was observed after three days. Importantly, the other disaccharides tested, sucrose and maltose, showed no response. These observations indicate that trehalose can serve as a trigger for *iniBAC* induction when it is taken up by the cell. To substantiate these results, we screened for transposon mutants that induced *iniBAC* induction at low trehalose levels. For this, we created another transposon mutant library in a WT *M*. *marinum* containing the integrated *iniBAC* reporter. Resulting mutants were plated on 2% trehalose, which only mildly induces a GFP signal in WT bacteria. Mutants that highly expressed *iniBAC* were identified by fluorescence microscopy, isolated and plated on 7H10 plates containing 2% trehalose and regular 7H10 plates to select for a trehalose-specific induction phenotype. In total, 25 mutants were found to be upregulated only on plates containing 2% trehalose. Strikingly, upon analysis of the transposon insertion sites by LM-PCR and sequencing, all mutations were mapped to the PDIM biosynthesis locus (Table 2). Our group has previously shown that PDIM mutations in *M*. *marinum* result in increased permeability of the mycobacterial outer membrane [20]. In conclusion, an increased outer membrane permeability by either introducing MspA or by disrupting PDIM biosynthesis results in increased *iniBAC* induction by the addition of trehalose.

**Table 2:** List of *M*. *marinum* mutants identified that showed high induction of *iniBAC* on 2% trehalose plates.

Gene ID	Gene product	H37Rv orthologue	Gene length	Unique mutants (bp from 5’ of gene)
*MMAR_1765*	FadD28	*Rv2941*	1743	223, 1009
*MMAR_1767*	Mas	*Rv2940c*	6300	154, 5071, 5266, 5654
*MMAR_1768*	PapA5	*Rv2939*	1245	1111
*MMAR_1772*	PpsE	*Rv2935*	4446	181, 1228, 1568, 1912, 4279, 4288
*MMAR_1773*	PpsD	*Rv2934*	5421	225, 4288
*MMAR_1774*	PpsC	*Rv2933*	6606	33, 484, 808, 3283
*MMAR_1775*	PpsB	*Rv2932*	4574	848, 3261
*MMAR_1777*	FadD26	*Rv2930*	1755	34, 254, 601
*MMAR_1777/MMAR_1778*	FadD26/TesA	*Rv2928/Rv2930*	1755/750	443 bp before 5' of *fadD26*

The H37Rv orthologue, if present, the gene length and the number of unique mutants per gene are indicated. Unique mutants are annotated by the position of the transposon insertion from the 5’ of the gene unless otherwise indicated

### Free trehalose accumulates in response to treatment with EMB, INH and BTZ

Previously, Winder and Brennan reported that INH-treated *M*. *smegmatis* accumulates free trehalose [25]. Because we show that addition of trehalose to mycobacterial cell can induce *iniBAC*, we hypothesized that addition of both EMB and INH, cause the accumulation of trehalose levels. In addition, we also included the drug candidate PBTZ169. BTZ has a similar effect as EMB, disrupting biosynthesis of the arabinogalactan layer by inhibiting DprE1 [6,7]. Because we did not know whether BTZ induces the *iniBAC* operon we first used our reporter assay to address this question. As can be seen in S3A Fig, 1 ng/ml BTZ caused a mild growth inhibition (S3A Fig). Subsequently, we used this concentration as well as a ten-fold dilution (0.1 ng/ml) to treat cultures of WT *M*. *marinum* containing our *iniBAC* reporter construct and measured fluorescence induction with flow cytometry. After addition of 1 ng/ml BTZ a high induction of fluorescence was observed over time, when compared to an untreated control (S3B–S3D Fig). To measure the effect of chemotherapeutic agents on free trehalose levels, we compared untreated cultures to cultures treated with either EMB, INH or BTZ and isolated trehalose with a hot water extraction after 3 and 6 hours. Cells were adjusted by OD600, as well as normalized to protein content. As shown in S4A Fig, there is a visible increase in the amount of free trehalose for INH, EMB and BTZ when compared with untreated cultures. However, the signal for INH was highest for both time points (S4B Fig).

Because we observed that an increase in permeability increased the ability of trehalose to induce *iniBAC* we assume that the trehalose enters from the outside into the periplasm. To address whether uptake of trehalose over the inner membrane is required for stress signal transduction, we used a transposon mutant, *sugA*::*tn*, that is defective in the only known inner membrane transporter of trehalose, *i*.*e*. the LpqY-SugA-SugB-SugC complex [26]. To measure whether induction is hampered in a *sugA*::*tn* mutant, we introduced our exosomal *iniBAC* reporter into this strain and assessed whether exogenous trehalose can still induce the *iniBAC* operon. We used a WT *M*. *marinum* with the exosomal stress reporter as a control and the gating strategy for our flow cytometry experiment can be found in S5A Fig. These experiments showed that the *sugA*::*tn* mutant can still readily induce *iniBAC* in response to 1% trehalose (S5B Fig). We also noticed that the operon was still inducible by EMB and INH (S5B Fig).When comparing the fold induction levels (corrected for the MFIs of the respective untreated controls) over time, the *sugA*::*tn* mutant induces *iniBAC* with at a comparable level as a WT *M*. *marinum*. On day 3 the *sugA*::*tn* mutant shows a higher fold induction (12.8 fold) compared with WT (9 fold), perhaps reflecting a higher concentration of periplasmic trehalose (S5C Fig). These combined results show that antimycobacterial compounds that target the cell wall can indeed lead to an increase in intracellular trehalose. We show that trehalose probably does not have to be transported across the cytoplasmic membrane, indicating that the sensing system required for induction is located in the periplasm.

### Purified IniR_Mtb_ forms multimers independent of trehalose

**Fig 10:**
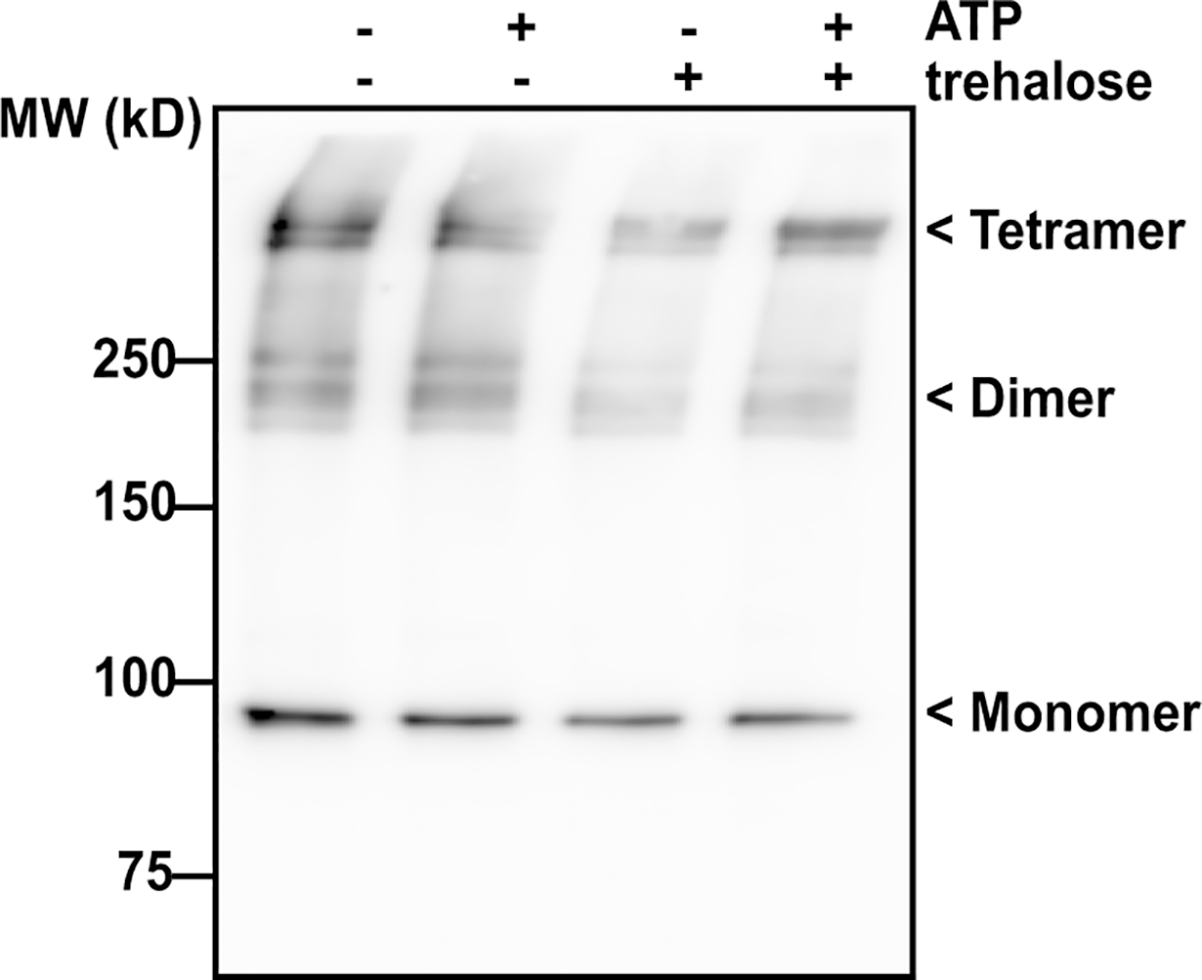
Immunoblot analysis of IniR_Mtb_ with α-Strep antibody. *M*. *smegmatis* containing the construct pEXCF-*iniR*_*Mtb*_-Strep vector was cultured and cultures were exposed for 18 hours to 10 ng/ml ATc/ Strep-tagged IniR_Mtb_ was isolated with StrepTactin beads. Purified protein from the elution fractions (elution 1–5, see S6 Fig) was exposed to either trehalose, ATP, both or neither. Presence is indicated with (+), absence is indicated with (-). Indicated on the image of the Western Blot are monomeric IniR (~90kDa) and possible dimers and tetramers.

Based on bioinformatics analysis (BLAST and Phyre2 [21]) we found that IniR contains two domains (the ATPase domain and the MalT domain III) that suggested possible multimerization. We hypothesized that trehalose could be required for multimerization, as maltose is required for MalT tetramerization in *E*. *coli* [23]. To address this, we constructed a Strep-tagged *iniR*_*Mtb*_ and cloned it into an ATc-inducible vector (to generate pEXCF-*iniR*_*Mtb*_-Strep). We transformed the plasmid into *M*. *smegmatis* and induced expression. Purified protein fractions were separated on an SDS-PAGE gel and stained with Coomassie (Elution fractions (1–5) can be found in S6 Fig). As shown in Fig 10, higher order structures can be identified in all tested conditions. The extra bands are approximately at the expected heights of dimers (180kDa) and tetramers (360kDa). Multimerization did not seem to be dependent on the exogenous addition of trehalose in this experimental setup. We also performed the experiment with a crude cell lysate of *M*. *smegmatis* that overexpressed FLAG-tagged IniR_Mtb_. However, also in this experiment we observed multimers independent of the presence of trehalose or ATP (S7 Fig). In summary, we see that IniR readily forms oligomeric structures similar to MalT in *E*. *coli* [23,24].

## Discussion

In this work, we describe the identification of the *iniBAC* regulator IniR, by performing transposon mutagenesis in a strain that highly expressed *iniBAC* by default. In our first publication on the regulation of the *iniBAC* operon we identified a strong link between upregulation of the *iniBAC* operon and the vitamin B12 dependent enzyme MutAB. This enzyme is a central component of the methylmalonyl-CoA pathway, a pathway that is involved in the degradation of fatty acids [13,14]. At the time, we attempted to identify a metabolic cue causing *iniBAC* activation. However, exogenously adding different kinds of fatty acids or small metabolites present or linked in this pathway did not influence *iniBAC* expression. In this work, trehalose was identified as a metabolic signal that activates the *iniBAC* operon. Moreover, increasing the permeability by introducing MspA (Fig 9B) or blocking PDIM production (Table 2) strongly increased the induction potency of trehalose. This finding also confirms our previous work that showed that PDIM mutations can allow for the essential uptake of nutrients when the ESX-5 system is deleted [20].

In mycobacteria, trehalose is used to synthesize a range of important outer membrane lipids, including trehalose monomycolates, trehalose dimycolates, penta-acyl trehaloses (PATs), diacyl trehaloses (DATs) and sulfolipids (only in members of the *M*. *tuberculosis* complex) [27,28]. Because of its importance, trehalose biosynthesis and metabolism has been extensively studied [29]. These studies showed that trehalose is recycled from TMMs, TDMs, PATs and DATs and import occurs via the LpqY-SugA-SugB-SugC system, identified and described Kalscheuer et al [26]. This system, upon knockout, causes attenuation of *M*. *tuberculosis in vivo*. Mycobacteria possess three separate biosynthesis routes for trehalose, a redundancy that also illustrates the importance of this disaccharide. The best studied route is the OtsA-OtsB pathway that synthesizes trehalose via glucose-6-phosphate and UDP-glucose intermediates [29]. Another pathway is the TreY-TreZ pathway that can synthesize trehalose from α(1→4)glucose polymers [30]. However, the glyoxylate shunt can also produce trehalose *de novo* from sucrose or maltose via the TreS pathway [26,31].

The most intriguing mutants linking *iniBAC* induction to trehalose are the multiple *mce1* operon transposon mutants. These mutants show repression of *iniBAC* in the highly expressing *cobC*::*tn* strain. The *mce1* operon, suggested to be involved in the salvage pathway mycolic acids, was reported to show a high accumulation of FMAs in *M*. *tuberculosis* [18]. We not only confirmed this finding for *M*. *marinum*, but also identified a strong decrease in bound mycolic acids, suggesting that *mce1* disruption has a pleiotropic effect in *M*. *marinum*. This multitude of effects of *mce1* disruption on the mycobacterial lipidome has also been illustrated in *M*. *tuberculosis* by previous work by Queiroz et al. who found that disruption of *mce1* significantly changes the abundance of over 400 lipid species [32]. An explanation for the decreased amounts of bound mycolic acids could be that the enzymatic reaction leading to increased amounts of FMAs is influenced by the amount of free mycolic acids. Another possible explanation comes from recent work by Ekiert et al., who provided the first structural insights into the function of the Mce proteins [33]. These authors showed that the *E*. *coli mce* homologues encode proteins that can from a hexameric complex that mediates lipid transport. They propose, based on their structural studies, that the mycobacterial *mce* orthologues may very well perform a similar function in lipid transport [33]. These structural predictions may explain the absence of FMA transport by *mce1* mutants. Without the shuttling of FMAs, the recycling of trehalose-containing lipids and the concomitant transport of free trehalose would also decrease. In line with this theory, we observed that the *mce1D*::*tn* mutant is still inducible with free trehalose (S2 Fig).

More evidence for links between TMM recycling and *iniBAC* induction comes from a recent paper by Degiacomi et al., who examined the effect of a knockdown of the essential inner-membrane TMM-flipase MmpL3 [34]. This procedure resulted in the upregulated expression of the *iniBAC* operon. Whether the induction is caused because of a lack of TMMs or an overflow of trehalose by breakdown of TMMs that are not flipped over the inner-membrane is unknown. These combined observations could also explain why *iniBAC* induction is observed when EMB and INH are used, in both cases there is a strong disruption of outer membrane biogenesis and a concomitant activation of the trehalose salvage pathway. Interestingly, we managed to confirm previous findings by Winder and Brennan who established that there is an accumulation of free trehalose as a result of isoniazid treatment [25]. We extended this observation to BTZ and EMB, further supporting that *iniBAC* induction could be mediated through an increase in trehalose.

The IniR protein contains three domains that together make it a member of the small family of signal-transduction ATPases with numerous domains, with a domain composition similar to MalT of *E*. *coli* (62%). In *E*. *coli* MalT is bound to the maltose uptake system and released upon the binding of maltose [23,24]. Binding maltotriose causes MalT to multimerize and leads to binding and transcription of the genes encoding the maltose transport system. For IniR the signaling molecule could be trehalose. These findings reveal trehalose as a unique cell wall stress signaling molecule that has not previously been described as such in gram negative bacteria. Gram negatives are known to activate their cell wall stress signaling cascades through the detection of mistranslated proteins or aberrant folding of membrane proteins, but not through disaccharide metabolites [35].

Future experiments should address the question whether iniR can sense or bind trehalose. Our data on the oligomerization of IniR at least suggests that it occurs in dimers and/or tetramers and that trehalose is not required for oligomerization of IniR. Understanding the interaction of IniR with trehalose or other binding partners will shed light on the function of the *iniBAC* operon and will allow us to address why IniR is so specific, regulating only the *iniBAC* genes. The discovery of IniR also sparks further speculation on IniBAC function. Both IniA and IniC contain the same ATPase domain (P-Loop NTPase domain) that is found also in the membrane-associated MalK, the ATP hydrolyzing unit of the maltose transporter that opens upon binding of maltotriose [36].

Studies on the localization of the IniBAC proteins and their association with IniR will allow us to address possible homology between the *E*. *coli* maltose uptake system and IniBAC. Our RNA sequencing data indicates that the stoichiometry of the system might favor a high amount of IniB molecules, as compared to the IniA and IniC counterparts of the operon. This high production of IniBAC proteins is also suggestive of a functional link between IniB, IniA and IniC, where all three are needed to mount an effective stress response. Another extrapolation of IniA and IniC protein function comes from the high similarity of the GTPase domain to dynamin family proteins. This may suggests that both proteins can function as vesiculation proteins, shielding the cell from possible toxic cell wall or lipid intermediates. Bacterial dynamins have already been shown to form tubulations in lipid bilayers in a GTP-dependent manner in *Nostoc punctiforme* [37]. The role of IniB in this process is unclear, especially because the presence and size of this protein is varying extensively between species. Sequence analysis indicates that the N-terminal part of IniB is highly conserved and possibly contains a domain that is required for interaction or secretion. Studying these *iniBAC* genes, but also other genes that are strongly induced by antibiotics may very well lead to the discovery of essential stress coping routes in mycobacteria that can be targeted by novel anti-TB therapeutics.

## Methods

### Bacterial strains, plasmids and culturing conditions

The *E*. *coli* strain ST08 (Clontech) was used to propagate plasmid DNA. Bacterial cultures were routinely grown at 37°C in LB broth with the addition of antibiotics kanamycin (25 μg/ml), hygromycin B (50 μg/ml) or streptomycin (30 μg/ml), where required. *M*. *marinum* wild type M^USA^, as described by Abdallah et al. [38], was used for the transposon mutagenesis experiments and generation of the *iniR* (*MMAR_0612*) knockout. Cultures were routinely grown in Middlebrook 7H9, supplemented with Middlebrook ADC and 0,05% Tween-80. *M*. *smegmatis* MC^2^155 was used for the modified reporter assay and cultured identically to *M*. *marinum*.

*M*.*tuberculosis* H37Rv WT and an isogenic *ΔRv0339c* mutant, generated using specialized transduction with a phage kindly provided by Michelle Larsen and Bill Jacobs, were used for RNA sequencing experiments and cultured in 7H9, supplemented with ADC, 0,05% Tween-80 and 0.2% glycerol [39,40]. Additional antibiotics isoniazid (Sigma) and ethambutol (Sigma) were added, where indicated, at mid-logarithmic phase. For isoniazid 10 μg/ml was routinely added, whereas for ethambutol 1 μg/ml was used (both written as 1x MIC [13]). For experiments with PBTZ169 1 ng/ml was used as 1x MIC. The PBTZ169 was provided by Stewart Cole (EPFL. Lausanne). For the modified reporter assay anhydrotetracycline, or ATc, (Sigma) was added to indicated final concentrations. For mycobacterial experiments on solid medium, Middlebrook 7H10 solid agar supplemented with Middlebrook OADC was used. Trehalose, maltose and sucrose were purchased from sigma and added to the final concentrations indicated in the figures. Both mycobacterial cultures and plates were grown at 30°C. All the strains that were used in this study, besides the ones derived from our transposon screens, can be found in S5 Table.

### Construction of plasmids

All the primers used in this study can be found in S3 Table. All plasmids used in this study can be found in S4 Table. Plasmids pSMT3*-iniB4-mEos3*.*1* and pMV-*iniB4-mEos3*.*1* were previously described by Boot et al. [13]. Plasmid pEXCF-*Rv0339c-FLAG* has been previously described in [41]. For clarity within this manuscript we refer to this construct as pEXCF-*iniR*_*Mtb*_-*FLAG* For complementation experiments, *iniR*_*Mm*_ (*MMAR_0612*) was amplified from *M*. *marinum* M^USA^ gDNA. A fragment of 2786 bp was generated with primers iniR_Mm_-Comp_FW and iniR_Mm_-Comp_RV containing the promoter and *iniR*_*Mm*_ gene. Primers were designed to include 15-bp overlapping regions with the target vector, a *pMV361* derivative described previously [13,42]. All PCRs were performed with iProof master mix (Bio-Rad). The pMV vector was digested with HindIII. Subsequently, InFusion (Clontech) was used according to manufacturer’s protocol to generate the complementation construct pMV-pr*iniR*_*Mm*_. The construct *pEXCF*-*IniR*_*Mtb*_*-FLAG* was digested with BsrGI and used for the introduction of *iniR*_*Mm*_. Primers iniR_Mm_-FLAG_FW and iniR_Mm_-FLAG_RV were used to amplify *iniR*_*Mm*_ from *M*. *marinum* M^USA^ gDNA with 15bp overlapping regions with the pEXCF vector backbone. The resulting construct, pEXCF*-iniR*_*Mm*_*-FLAG* was generated with InFusion reagents. The plasmid containing *iniR*_*Mtb*_*-Strep* (*Rv0339c*) was constructed similarly. pEXCF- *iniR*_*Mtb*_*-FLAG* was digested with BsrGI. Subsequently *iniR*_*Mtb*_ was amplified with primers iniR_Mtb_-Strep-FW and iniR_Mtb_-Strep-RV, containing a strep-tag in a first round of PCR. This product was purified and used as a template for primers with a 15bp overhang (iniR_Mtb_-BsrGI-FW and iniR_Mtb_-BsrGI-RV) with the pEXCF vector backbone. This product was again purified and ligated into the digested pEXCF backbone with InFusion, resulting in construct pEXCF- *iniR*_*Mtb*_ *-Strep*

### Mycobacterial lipids extraction and TLC analysis

The selected transposon mutants *cobC*::*tn*, *cobC*::*tn*+*mce1D*, *cobC*::*tn*+*yrbE1B*::*tn* and *cobC*::*tn*+*cmaA2*::*tn* and a WT *M*. *marinum* M^USA^ were pre-cultured, diluted and grown to an OD600 of 1.0. Subsequently, 50 OD units were collected by centrifugation and the resulting pellets were washed three times with PBS. The pellets were weighed and adjusted accordingly to provide equal pellet masses. Subsequently, extraction of the cell envelope lipids was performed in three steps to harvest different lipid fractions: the apolar, polar and mycolic acids as previously described by Carrère-Kremer et al. and Minnikin et al. [43,44]. Both FMAs and bound mycolic acids were analyzed by 1D-TLC. For each sample, equal amounts of lipid fractions were loaded onto a silica-60 TLC plate (Merck) and separated by 1D-TLC. For MAMEs and FAMEs analysis running solvent hexane:ethylacetate 19/1 (v/v) was used. FMA analysis running solvent hexane:di-ethyl ether:HAc 70/30/1 (v/v/v) was used (as previously described by Ritu Bansal-Mutalik and Hiroshi Nikaido [45]. Subsequently, the lipids were visualized with 5% molybdophosphoric acid (MPA) in methanol and TLC-plate charring at 160°C for 10 minutes.

### Determination of the minimal inhibitory concentration (MIC) for antibiotics

For MIC assays *M*. *marinum* M^USA^ WT and the indicated transposon mutants were grown in 7H9 supplemented with ADC to an OD600 of 0.6–1, containing the appropriate antibiotics. Serial two-fold dilutions of ciprofloxacin, ethambutol, isoniazid, streptomycin and rifampicin (all from Sigma) were added per well in a 96-well plate. Per well, 10^4^ bacterial cells were added. The 96-wells plate was inoculated for 7 days before the minimal inhibitory concentration (MIC) was determined. This concentration is described as the concentration of antibiotics that showed visible growth inhibition. Wells without the addition of antibiotics and 7H9 medium itself were used as a growth control and experiments. All the growth assays were performed in triplicate.

### Ethidium bromide uptake assay in microtiter plates

The uptake of ethidium bromide in transposon mutants *cobC*::*tn* and *mce1D*::*tn* + *cobC*::*tn* was compared to WT *M*. *marinum* M^USA^. As a positive control, we overexpressed *mspA* in WT *M*. *marinum* M^USA^ using plasmid pSMT3*-hsp60-mspA* [20]. Strains were grown in 7H9 with ADC and 0.02% tyloxapol, cell were washed with PBS containing 0.02% tyloxapol and diluted to OD600 of 1.0. Then 180 μl of bacterial cells were distributed per well in a 96-wells microtiter plate. Subsequently, ethidium bromide was added to a final concentration of 5 μg/ml. The fluorescence was determined at 30°C using a plate reader (Biotek, excitation: 300 nm, emission: 605 nm, bottom-reading mode). Measurements were performed in quadruplicate.

### Transposon mutagenesis

Transposon mutagenesis was performed in *M*. *marinum* M^USA^ containing pMV-*iniB4-mEos3*.*1* The strain was infected with the mycobacterial phage phiMycoMarT7 that contains the *Himar1* transposon with kanamycin resistance, as described previously by Sassetti et al. [46,47]. Transposon mutants were assessed for increased mEos3.1 expression, indicating induction of *iniBAC*, manually by fluorescence microscopy. For the permeability mutagenesis screen, resulting mutants were plated on 7H10 plates containing 2% trehalose. Colonies that showed a vast increase in fluorescence as assessed by fluorescence microscopy were isolated and the transposon insertion site was determined by ligation-mediated PCR (LM-PCR) as described previously [38]. A *cobC*::*tn* mutant was used for a second round of mutagenesis, this time with a Himar transposon containing a hygromycin resistance cassette [46,47]. Colonies that showed no or severely decreased mEos3.1 expression on 7H10 plates were selected manually by fluorescence microscopy. These colonies were streaked on fresh 7H10 plates and compared to WT *M*. *marinum* containing the reporter construct for comparison of fluorescence. Mutants that showed no *iniBAC* expression were isolated and LM-PCR was used to identify the transposon insertion site, utilizing the *cobC*::*tn* parent strain as a control.

### Flow cytometry analysis

The induction of the *iniBAC* was routinely assessed by flow cytometry on a BD Accuri C6 flow cytometer (BD biosciences). For induction experiments bacteria were grown to a mid-log phase and diluted to an OD of 0.2. Subsequently, antibiotics or disaccharides were added at indicated concentrations for a specified amount of time. Time point analysis was performed by sampling 1 ml of culture. The sample was washed in PBS containing 0.05% Tween-80. The bacteria were then spun down and the resulting bacterial pellet was in PBS containing 0.05% Tween-80. Flow cytometry analysis was performed with a 488 nm laser and 530/30 nm filter for mEos3.1. Per sample, 30.000 gated events were analyzed per sample per time point and data was analyzed and visualized using BD CFlow software and Graphpad Prism 6. For samples the mean fluorescence intensity (MFI) was used to quantify fluorescence intensity.

### Generation of an Δ*iniR* mutant in *M*. *marinum*

The *iniR*_*Mm*_ (*MMAR_0612*) knockout was created via allelic exchange with the phAE159 temperature-sensitive phage method in *M*. *marinum* M^USA^. Details on the origin of the phage system can be found in the manuscript by Jain et al. and further details on the method can be found in Phan et al. [40,48]. The knockout construct was generated by PCR by amplification of left (iniR_Mm__L-FW+ iniR_Mm__L_RV) and right flanking (iniR_Mm__R_FW + iniR_Mm__R_RV) regions of *iniR*_*Mm*_, covering a total of 82% of the gene (see S1 Table). The deletion was confirmed by PCR analysis and sequencing. Subsequently, the hygromycin resistance cassette was removed by γδ-resolvase (TnpR) and counter-selected with SacB for sucrose sensitivity.

### IniR multimerization assay and immunoblot analysis

An *M*. *smegmatis* strain containing the plasmid with pEXCF-*iniR*_*Mtb*_-FLAG was grown in 7H9 liquid medium supplemented with ADC, 0.05% Tween-80 until mid-logarithmic phase, after which the cells were washed and inoculated in 7H9 medium with or without 10 ng/ml ATc to an OD600 of 0.2 and grown for another 18 h. Subsequently, bacterial cultures were spun down for 10 min at 6,000 × g, washed with phosphate-buffered saline (PBS), and resuspended in PBS to a concentration of 20 OD units/ml. Cells were lysed by bead-beating with 0.1mm silica beads 3 times for 20 seconds each. Then 25 mM MgSO_4_ was added, along with DNaseI (Thermo Fischer), to degrade chromosomal DNA and incubated for 1 hour at 37 degrees Celsius. Samples were spun down for 15 minutes at 16.000 x g to isolate soluble proteins. Subsequently, samples were split in equal volumes and trehalose (1%) and/or ATP (to 1 mM) were added to the appropriate samples. The resulting mixture was crosslinked with 1% formaldehyde for 30 minutes, on ice and quenched with 100 mM cold glycine for 30 minutes. The purified IniR_Mtb_-Strep protein was acquired by expressing the ATc-inducible vector containing pEXCF- *iniR*_*Mtb*_*-Strep* to 10 ng/ml ATc for 18 hours. 50ml cultures were spun down and cell pellets were resuspended in 100 mM Tris-HCl pH 8.0, 150 mM NaCl. The cells were lysed at 1 kilobar with the Stansted cell homogenizer. Unbroken cells were removed by centrifugation (3000 x g), after which the lysate was centrifuged for 45 minutes at 200.000 x g to remove insoluble cell components. Strep-tagged IniR_Mtb_ was purified from the soluble fraction by StrepTactin beads (iba-lifesciences) and eluted with 10 mM desthiobiotin, 10% glycerol, 50 mM Tris-HCl pH 8.0 and 150 mM NaCl. The protein fractions were separated on any kD SDS-PAGE gels (BioRad) and transferred to a nitrocellulose membrane. Membranes were stained with anti-FLAG M2 (Abcam) antibodies or anti-Strep-tag-II (Abcam) The secondary antibody was a goat anti-mouse antibody coupled to a peroxidase (Abcam) as secondary antibody for the anti-FLAG M2 and a goat anti-rabbit antibody coupled to a peroxidase (Abcam) as secondary antibody for the anti-Strep antibody. Nitrocellulose blots were developed with ECL Western Blotting substrate (Pierce) and chemiluminescence was visualized with a Amersham 600 imager (GE healthcare).

### Chromatin immunoprecipitation (ChIP) sequencing on IniR_Mtb_

ChIP was done following protocols previously used (PMID: 25581030 and PMID: 23823726) from [41]. Briefly, 50 ml of log phase *M*. *tuberculosis* (containing pEXCF-*iniR*_*Mtb*_-FLAG) culture was cross-linked with 1% formaldehyde for 30 minutes, then quenched with 250 mM glycine. Cells were pelleted, washed in PBS with protease inhibitor (Sigma) and resuspended in ChIP buffer (20 mM HEPES pH 7.9, 50 mM KCl, 0.5 mM DTT and 10% glycerol) with protease inhibitor. Samples were lysed in Lysing Matrix B tubes with three rounds of bead beating at maximum speed for 30 s. Beads were pelleted, supernatants removed, and ChIP buffer added to a final volume of 500 μl. A Covaris S2 ultrasonicator was used to shear chromatin to ~200 bp fragments. Samples were adjusted to buffer IPP150 (10 mM Tris-HCl—pH 8.0, 150 mM NaCl and 0.1% NP40) and immuno-precipitated by incubating samples overnight rotating at 4°C with 10 mg M2 anti-FLAG antibody. Samples were then incubated with protein G-coupled agarose beads for 30 min at 4°C and then 90 min at room temperature. Samples were pelleted for 2min at 2,000xg, supernatant discarded, washed five times in IPP150 buffer, then twice with TE, pH 8.0. Protein complexes were eluted off the beads with elution buffer (50 mM Tris-HC pH 8.0, 10 mM EDTA and 1% SDS) for 15 min at 65°C. Eluted protein–bead complexes were treated with TE pH 8.0 and 1% SDS for 5min at 65°C, then digested and de-crosslinked with 1 mg/ml Pronase for 2 h at 42°C followed by 9 h at 65°C. Finally, immuno-precipitated DNA was purified using QiaQuick PCR columns.

### RNA sequencing on H37Rv and H37Rv*-ΔRv0339c*

Transcriptional RNA profiles between H37Rv wild type and a *iniR*_*Mtb*_ (*ΔRv0339c*) were compared in triplicate. Cultures were grown until an OD600 of 0.2 and at that point INH or EMB was added to the cultures and allowed to respond to drug stress for 24 hours. Cultures without antibiotic treatment served as a control. Subsequently RNA was isolated using standard methods described previously [49] and rRNA was depleted using RiboZero Gold (Illumina) RNA sequencing was done using Illumina Nextseq with libraries generated using the NEBNext directional library kit (NEB). Alignment to the H37Rv genome was done using Bowtie 2.0 [50], read depth was determined using DuffyNGS tools (available at https://github.com/sturkarslan/DuffyNGS), and visualized using Arraystar (DNASTAR).

### Mycobacterial trehalose extraction and TLC analysis

For isolation of trehalose from WT *M*. *marinum* cells were grown in 7H9 containing 0.2% glycerol, ADC and 0.05% Tween-80. Subsequently, cells were washed thrice with 7H9 containing 0.2% glycerol and 0.05% Tween-80 and diluted to OD 0.1 and split into 50 ml cultures (again in 7H9 containing 0.2% glycerol and 0.05% Tween-80). Antibiotics were added to final concentrations of 1 ng/ml for pBTZ169, 1 μg/ml for EMB and 10 μg/ml for INH. An untreated control was taken along for reference. Directly after inoculation, one culture per tested condition was spun down at 4.000 x g and the pellet was washed with PBS and stored at -20°C. This was time point was considered 0 hours. Subsequently, after 3 hours and 6 hours cultures were sacrificed and pellets were isolated, washed with PBS. The dry pellets were further adjusted on a microbalance according to pellet weight. Subsequently bacterial pellets were resuspended in distilled H_2_O(20 OD units/ml). To further normalize samples, protein content was measured and equalized for each sample before loading it on TLC plates. To this end, 250 μL of cell suspension was mixed with glass beads (0.1 mm diameter, zirconia/silica) and cells were disrupted with a beadbeater for 1 min. After briefly settling the beads (by leaving them for 1 min at RT), the protein content of the supernatant was determined using a BCA protein assay kit (Pierce). The free sugars of the bacterial cells were extracted by boiling of 250 μL of the pellet suspension in water at 99°C for 10 min. Afterwards, the cellular debris were removed by centrifugation at 16,000 x g for 5 min. The supernatant containing the hot water extracts was transferred into new tubes and normalized according to protein content as measured with the BCA protein assay kit. Hot water extracts were spotted on TLC plates (HPTLC, silica gel 60) and addition of trehalose and glucose served as reference (1 μl of a 1 mM solution of both was spotted). The sugars were separated using the solvent system acetonitrile–water (7:3). The sugars were visualized by spraying anthrone solution (10 mM anthrone in concentrated sulfuric acid) and subsequent charring at 110°C until the controls became visible. The quantification of the trehalose signals were calculated using GelQuant.NET V1.7.8 (available at http://www.biochemlabsolutions.com). Intensity of the trehalose signals were corrected for background and the intensities of treated samples were divided to their corresponding untreated time point controls to get fold change intensities.

## Supporting information

S1 Fig There are no permeability differences between the *mce1::tn* on/off mutant and the *cobC::tn* parent strain. An ethidium bromide (EtBr) uptake assay was used to compare permeability of WT *M*. *marinum*, the *cobC*::*tn* parent mutant and the *mce1D*::*tn* + *cobC*::*tn* double mutant. A WT *M*. *marinum* that expresses porin MspA was used as a positive control. The EtBr uptake was followed over the course of two hours in a microtiter plate reader. Fluorescence intensity (a.u.) is depicted on the Y-axis. There are no major differences in EtBr uptake for the *cobC*::*tn* parent and *mce1D*::*tn* + *cobC*::*tn* double mutant. The experiment was performed with a biological quadruplicate, error bars indicate the s.d.
(TIF)

S2 Fig Trehalose can still induce *iniBAC* in an *mce1::tn* on/off mutant. Addition of trehalose induces *iniBAC* transcription in *mce1* mutant background as measured by flow cytometry. Induction with trehalose of the *cobC*::*tn* (black) parent strain was compared to *cobC*::*tn+mce1D*::*tn* (dark grey) and *cobC*::*tn+cmaA2*::*tn* (light grey) by exposing cultures to 1% trehalose and measuring mean fluorescence intensity over time with a flow cytometer. For day 1, 2 and 3 the MFI of 1% trehalose treated cultures was divided by the untreated controls, leading to fold induction values. Data is from a biological triplicate. Error bars represent s.d. values.
(TIF)

S3 Fig Benzothiazinones (PBTZ169) strongly induce *iniBAC*. BTZ (PBTZ169) was tested at 1x MIC (1 ng/ml) and at 0.1x MIC (0.1 ng/ml) on *M*. *marinum* WT containing our *iniBAC* reporter construct to observe growth. (A) OD600 measured over time for 1x MIC and 0.1x MIC. There is a slight growth defect of the 1x MIC concentration, indicating that the cells are stressed. (B) The gating strategy of flow cytometry experiments. The gate was drawn to select a population that is roughly equal in size and granularity (side scatter, SSC and forward scatter, FSC). The gated population was used for all samples. A total of 30,000 cells were analyzed per sample. (C) The histograms 3 days after treatment with 1x MIC (1 ng/ml in blue) or 0.1x MIC (0.1 ng/ml in orange) compared to an untreated sample of *M*. *marinum* containing the reporter construct. Fluorescence intensity of mEos3.1 is measured in arbitrary units. Data is representative of one experiment from a triplicate of independent experiments. 1x MIC BTZ clearly induces *iniBAC* as measured by flow cytometry. (D) Quantification of the biological triplicate of samples over time for untreated *M*. *marinum* (black bars), compared to 1x MIC (blue bars) and 0.1x MIC (orange bars).
(TIF)

S4 Fig Treatment with INH, EMB and BTZ cause an accumulation of free trehalose. To address whether trehalose accumulates upon treatment with isoniazid (INH), ethambutol (EMB) and benzothiazinone (BTZ), we performed thin layer chromatography (TLC) experiments. In (A) *M*. *marinum* cultures were grown in 7H9 with glycerol and 0.05% Tween-80 and exposed to 1x MIC INH (10 μg/ml), 1x MIC EMB (1 μg/ml) or 1x MIC BTZ (1 ng/ml) for 3 hours and 6 hours. Trehalose was extracted and spotted on glass TLC plates. An untreated control was taken along as well. Glucose and trehalose were spotted as a reference (1 μl of a 1 mM solution). The experiment was performed in triplicate. In (B) a quantification of the triplicate of experiments is depicted. Fold change was calculated (with GelQuant V1.7.8) by dividing the intensity of the trehalose band of treated conditions to their respective untreated time point control.
(TIF)

S5 Fig Periplasmic trehalose can induce *iniBAC* transcription. By disrupting the only known trehalose transporter system LpqY-SugA-SugB-SugC in a *sugA*::*tn* mutant, we examined the origin of the *iniBAC* induction signal. In (A) The gating strategy of flow cytometry experiments. The gate was drawn to select a population that is roughly equal in size and granularity (side scatter, SSC and forward scatter, FSC). The gated population was used for all samples. A total of 30,000 cells were analyzed per sample. (B) Histograms of the fluorescence induction (mEos3.1 fluorescence intensity in arbitrary units) of the *iniBAC* reporter in a WT *M*. *marinum* (left panel) as well as a *sugA*::*tn* mutant (right panel) following treatment with 1%t trehalose (blue line), EMB (red line) or INH (orange line). Similar induction patterns can be observed for both strains. The histograms are representative of an experiment performed in triplicate. (C) Quantification of the average fold inductions of three independent experiments. The fold induction was calculated by dividing the MFI of the treated sample to the MFI of the corresponding untreated control.
(TIF)

S6 Fig Purified IniRMtb-Strep after using StrepTactin beads. After purification with StrepTactin beads, the elution fractions (E1-E5), loaded sample before purification (L) and flow-through (FT) were separated on an SDS-PAGE gel. Coomassie staining was used to visualize proteins. A page-ruler prestained protein ladder was ran as marker (PR). Elution fractions E1-E5 contain highly purified fractions of IniR_Mtb_. Monomeric IniR_Mtb_ runs around 90 kDa and is indicated with the ‘<‘.
(TIF)

S7 Fig Immunoblot analysis of IniR-TB with α-FLAG antibody. *M*. *smegmatis* cultures were exposed for 18 hours to 10 ng/ml ATc and soluble proteins were isolated. Fractions were exposed in the presence (+) or absence (-) of trehalose and/or ATP and subsequently crosslinked with formaldehyde (+) or not (-). Indicated on the Western Blot are monomeric IniR (~90kDa), possible dimers and tetramers.
(TIF)

S1 Table MIC determination on *iniR_Mm_* mutant shows no change in antibiotic susceptibility. Measurements were performed in triplicate. The concentration at which no visible growth was seen is denoted as the MIC value in μg/ml. The standard deviation is shown between brackets behind the values in the table. WT is *M*. *marinum* M^USA^.
(DOCX)

S2 Table Fold changes in expression of *iniBAC* in *M. tuberculosis*. The fold changes in expression between H37Rv and the *ΔiniR* mutant were calculated for *iniR*, *iniB*, *iniA* and *iniC* for untreated, ethambutol treated and isoniazid treated samples. To do so the log2RPKM values of the H37Rv wild-type samples were divided by the log2RPKM values of the *ΔiniR* mutant.
(DOCX)

S3 Table Primers used in this study. (DOCX)

S4 Table List of plasmids used in this study. (DOCX)

S5 Table List of strains used this study. (DOCX)
