# Supplementary figures and images for: Cell envelope stress in mycobacteria is regulated by the novel signal transduction ATPase IniR in response to trehalose
Source: PLoS Genet. 2017 Dec 27;13(12):e1007131. doi: 10.1371/journal.pgen.1007131 (PMC5760070; doi:10.1371/journal.pgen.1007131)

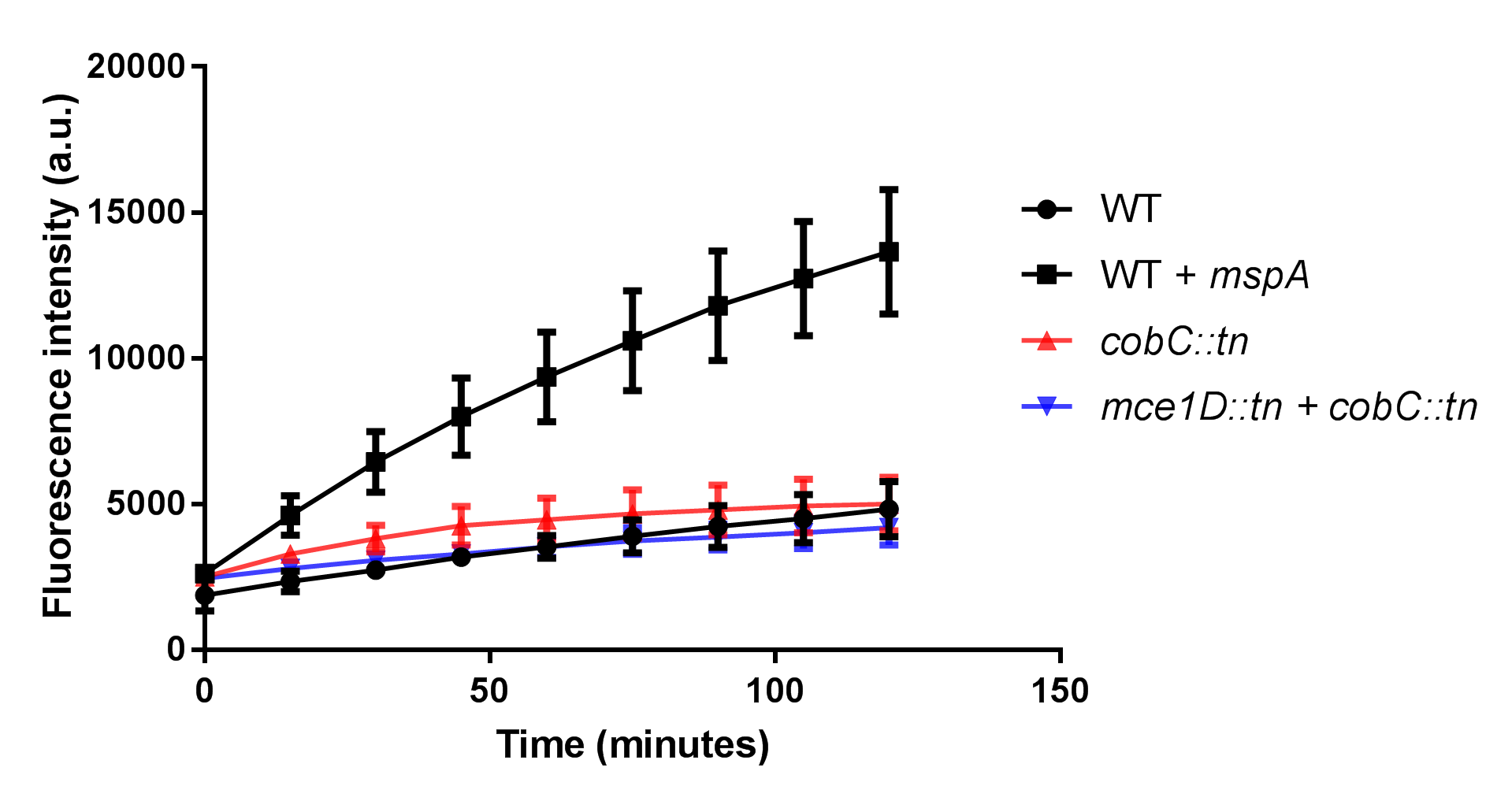

Supplement: S1 Fig — An ethidium bromide (EtBr) uptake assay was used to compare permeability of WT M. marinum, the cobC::tn parent mutant and the mce1D::tn + cobC::tn double mutant. A WT M. marinum that expresses porin MspA was used as a positive control. The EtBr uptake was followed over the course of two hours in a microtiter plate reader. Fluorescence intensity (a.u.) is depicted on the Y-axis. There are no major differences in EtBr uptake for the cobC::tn parent and mce1D::tn + cobC::tn double mutant. The experiment was performed with a biological quadruplicate, error bars indicate the s.d. (TIF) [file pgen.1007131.s001.tif]

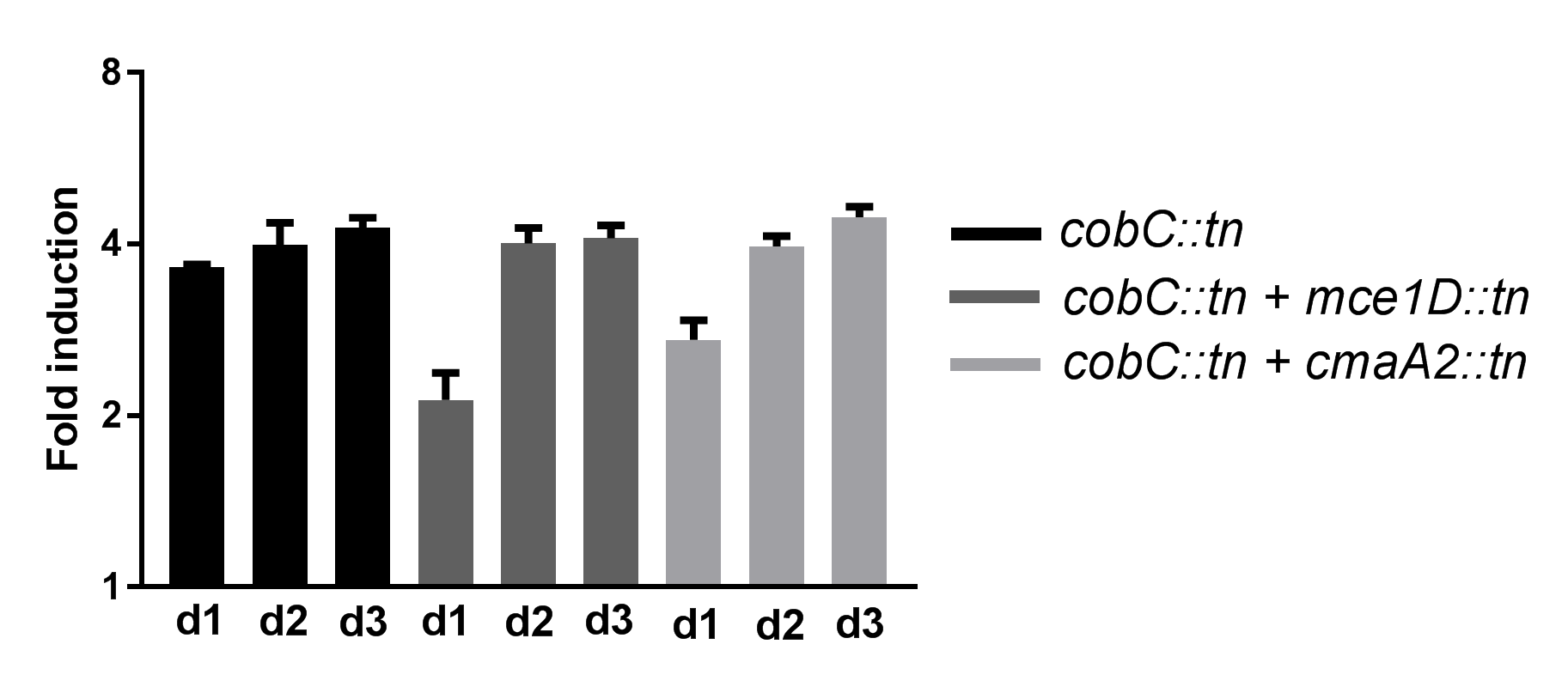

Supplement: S2 Fig — Addition of trehalose induces iniBAC transcription in mce1 mutant background as measured by flow cytometry. Induction with trehalose of the cobC::tn (black) parent strain was compared to cobC::tn+mce1D::tn (dark grey) and cobC::tn+cmaA2::tn (light grey) by exposing cultures to 1% trehalose and measuring mean fluorescence intensity over time with a flow cytometer. For day 1, 2 and 3 the MFI of 1% trehalose treated cultures was divided by the untreated controls, leading to fold induction values. Data is from a biological triplicate. Error bars represent s.d. values. (TIF) [file pgen.1007131.s002.tif]

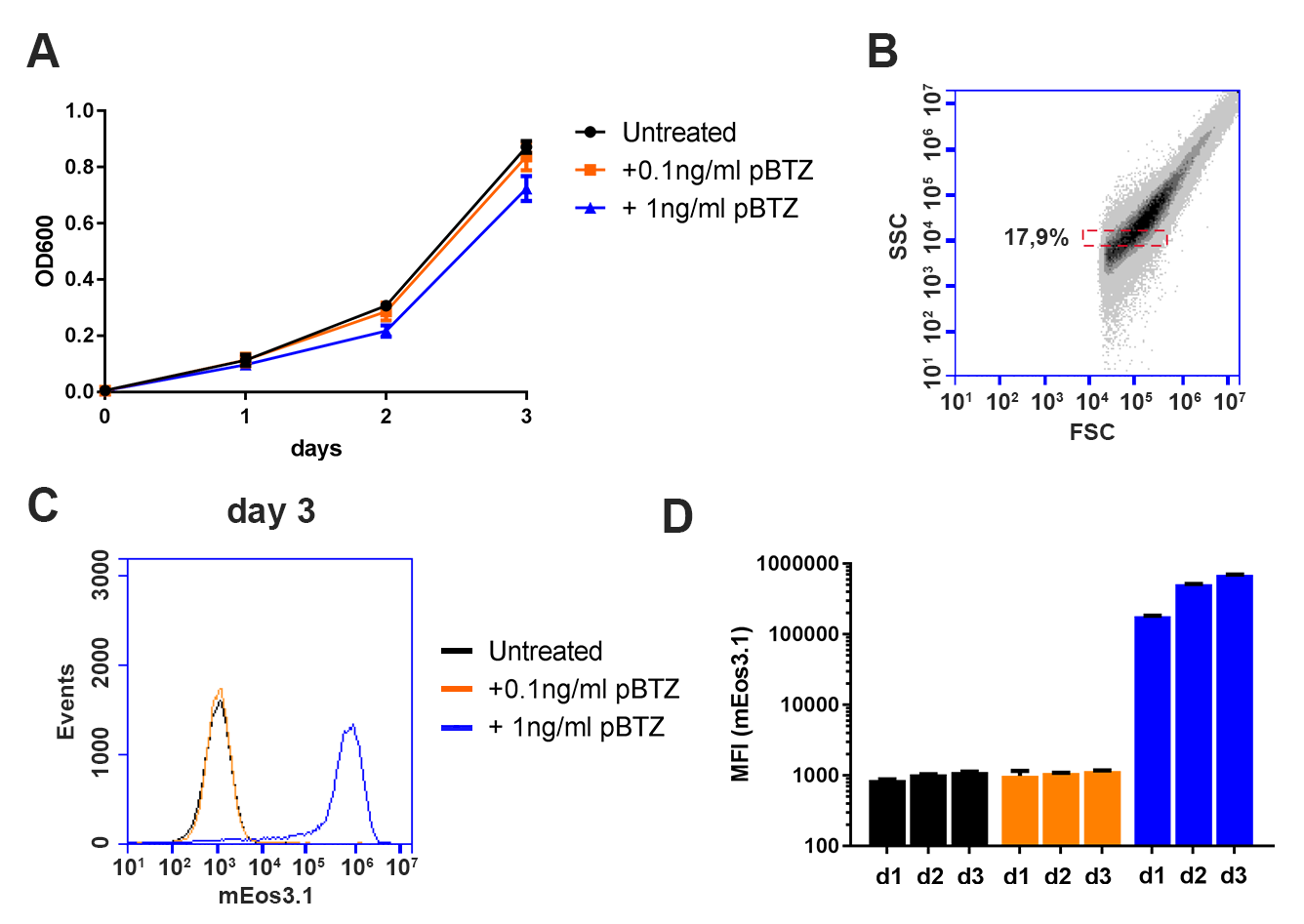

Supplement: S3 Fig — BTZ (PBTZ169) was tested at 1x MIC (1 ng/ml) and at 0.1x MIC (0.1 ng/ml) on M. marinum WT containing our iniBAC reporter construct to observe growth. (A) OD600 measured over time for 1x MIC and 0.1x MIC. There is a slight growth defect of the 1x MIC concentration, indicating that the cells are stressed. (B) The gating strategy of flow cytometry experiments. The gate was drawn to select a population that is roughly equal in size and granularity (side scatter, SSC and forward scatter, FSC). The gated population was used for all samples. A total of 30,000 cells were analyzed per sample. (C) The histograms 3 days after treatment with 1x MIC (1 ng/ml in blue) or 0.1x MIC (0.1 ng/ml in orange) compared to an untreated sample of M. marinum containing the reporter construct. Fluorescence intensity of mEos3.1 is measured in arbitrary units. Data is representative of one experiment from a triplicate of independent experiments. 1x MIC BTZ clearly induces iniBAC as measured by flow cytometry. (D) Quantification of the biological triplicate of samples over time for untreated M. marinum (black bars), compared to 1x MIC (blue bars) and 0.1x MIC (orange bars). (TIF) [file pgen.1007131.s003.tif]

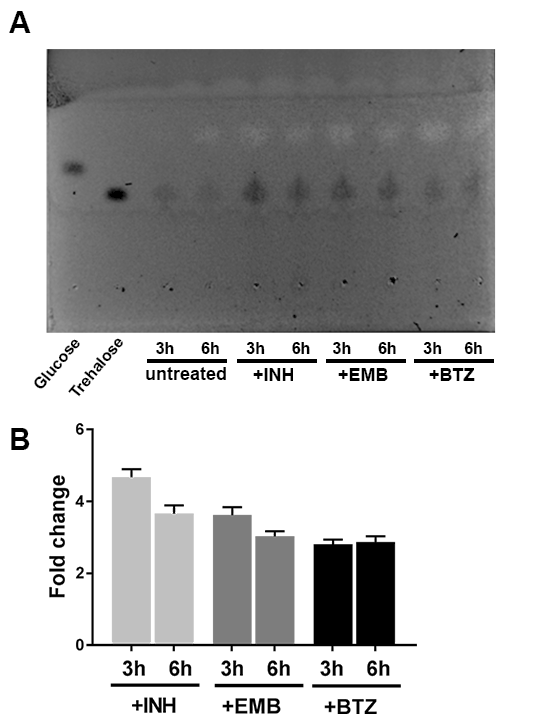

Supplement: S4 Fig — To address whether trehalose accumulates upon treatment with isoniazid (INH), ethambutol (EMB) and benzothiazinone (BTZ), we performed thin layer chromatography (TLC) experiments. In (A) M. marinum cultures were grown in 7H9 with glycerol and 0.05% Tween-80 and exposed to 1x MIC INH (10 μg/ml), 1x MIC EMB (1 μg/ml) or 1x MIC BTZ (1 ng/ml) for 3 hours and 6 hours. Trehalose was extracted and spotted on glass TLC plates. An untreated control was taken along as well. Glucose and trehalose were spotted as a reference (1 μl of a 1 mM solution). The experiment was performed in triplicate. In (B) a quantification of the triplicate of experiments is depicted. Fold change was calculated (with GelQuant V1.7.8) by dividing the intensity of the trehalose band of treated conditions to their respective untreated time point control. (TIF) [file pgen.1007131.s004.tif]

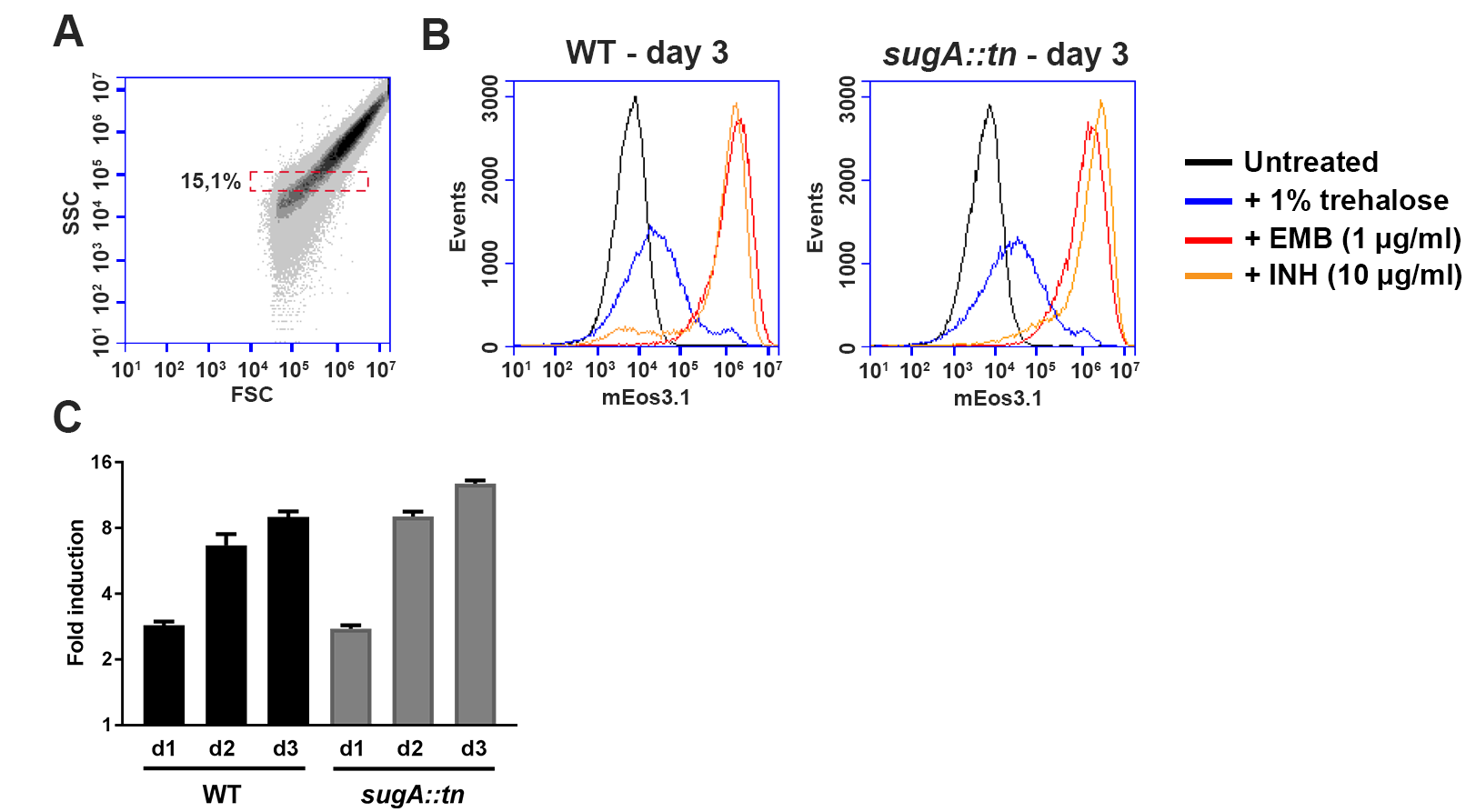

Supplement: S5 Fig — By disrupting the only known trehalose transporter system LpqY-SugA-SugB-SugC in a sugA::tn mutant, we examined the origin of the iniBAC induction signal. In (A) The gating strategy of flow cytometry experiments. The gate was drawn to select a population that is roughly equal in size and granularity (side scatter, SSC and forward scatter, FSC). The gated population was used for all samples. A total of 30,000 cells were analyzed per sample. (B) Histograms of the fluorescence induction (mEos3.1 fluorescence intensity in arbitrary units) of the iniBAC reporter in a WT M. marinum (left panel) as well as a sugA::tn mutant (right panel) following treatment with 1%t trehalose (blue line), EMB (red line) or INH (orange line). Similar induction patterns can be observed for both strains. The histograms are representative of an experiment performed in triplicate. (C) Quantification of the average fold inductions of three independent experiments. The fold induction was calculated by dividing the MFI of the treated sample to the MFI of the corresponding untreated control. (TIF) [file pgen.1007131.s005.tif]

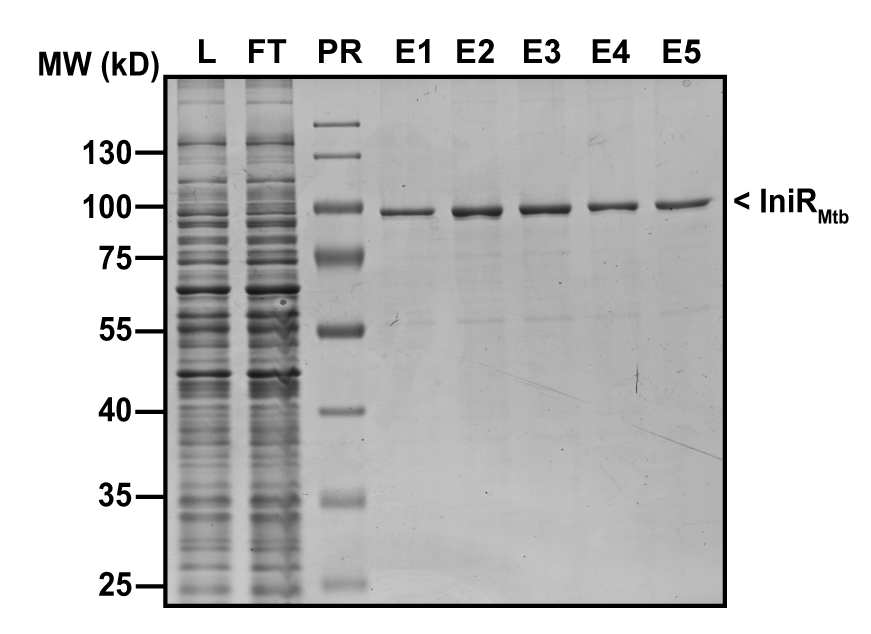

Supplement: S6 Fig — After purification with StrepTactin beads, the elution fractions (E1-E5), loaded sample before purification (L) and flow-through (FT) were separated on an SDS-PAGE gel. Coomassie staining was used to visualize proteins. A page-ruler prestained protein ladder was ran as marker (PR). Elution fractions E1-E5 contain highly purified fractions of IniRMtb. Monomeric IniRMtb runs around 90 kDa and is indicated with the ‘<‘. (TIF) [file pgen.1007131.s006.tif]

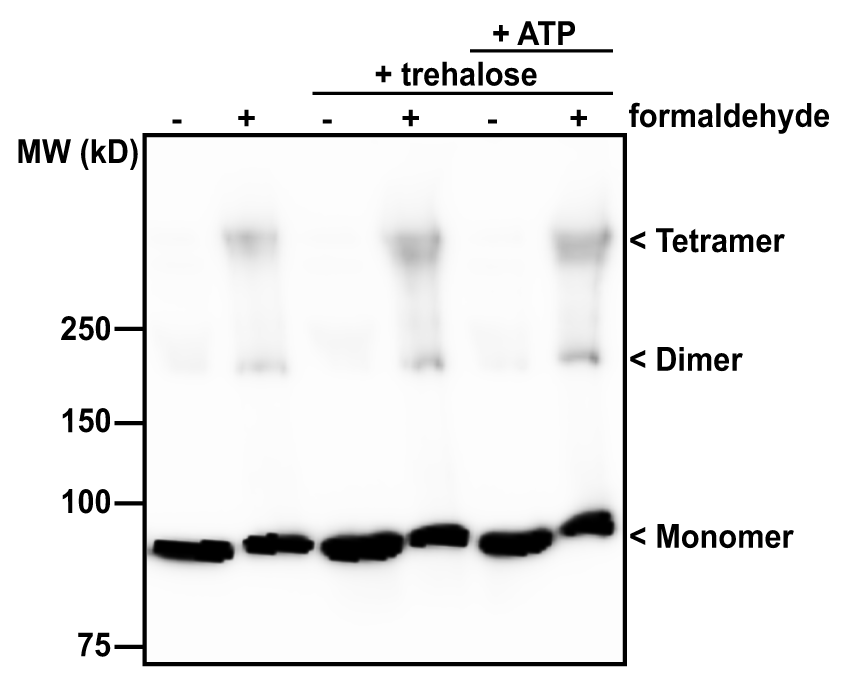

Supplement: S7 Fig — M. smegmatis cultures were exposed for 18 hours to 10 ng/ml ATc and soluble proteins were isolated. Fractions were exposed in the presence (+) or absence (-) of trehalose and/or ATP and subsequently crosslinked with formaldehyde (+) or not (-). Indicated on the Western Blot are monomeric IniR (~90kDa), possible dimers and tetramers. (TIF) [file pgen.1007131.s007.tif]
