# Supplementary material for: Cell envelope stress in mycobacteria is regulated by the novel signal transduction ATPase IniR in response to trehalose
Source: PLoS Genet. 2017 Dec 27;13(12):e1007131. doi: 10.1371/journal.pgen.1007131 (PMC5760070; doi:10.1371/journal.pgen.1007131)
Supplement: S1 Table — Measurements were performed in triplicate. The concentration at which no visible growth was seen is denoted as the MIC value in μg/ml. The standard deviation is shown between brackets behind the values in the table. WT is M. marinum MUSA. (DOCX) [file pgen.1007131.s008.docx]

S1 Table

| Strain | Rifampicin (µg/ml) | Ethambutol (µg/ml) | Isoniazid (µg/ml) | Ciprofloxacin (µg/ml) |
| --- | --- | --- | --- | --- |
| WT | 0.4 | 0.6 | 16.7 (7.2) | 0.2 |
| *cobC::tn* | 0.4 | 0.6 | 12.5 | 0.2 |
| *cobC::tn+ mce1D::tn* | 0.33 (0.11) | 1.25 | 50 | 0.2 |
| *cobC::tn+ yrbE1B::tn* | 0.17 (0.06) | 0.6 | 25 | 0.2 |
| *cobC::tn+ sdhA1::tn* | 0.8 | 0.65 | 25 | 0.4 |
| *cobC::tn+ MMAR_0612* | 0.8 | 0.65 | 25 | 0.4 |
