# Supplementary material for: Cell envelope stress in mycobacteria is regulated by the novel signal transduction ATPase IniR in response to trehalose
Source: PLoS Genet. 2017 Dec 27;13(12):e1007131. doi: 10.1371/journal.pgen.1007131 (PMC5760070; doi:10.1371/journal.pgen.1007131)
Supplement: S2 Table — The fold changes in expression between H37Rv and the ΔiniR mutant were calculated for iniR, iniB, iniA and iniC for untreated, ethambutol treated and isoniazid treated samples. To do so the log2RPKM values of the H37Rv wild-type samples were divided by the log2RPKM values of the ΔiniR mutant. (DOCX) [file pgen.1007131.s009.docx]

S2 Table

| Condition/  Gene name | Untreated | INH | EMB |
| --- | --- | --- | --- |
| *iniR* | 4,6 | 3,2 | 3,6 |
| *iniB* | 15,7 | 357,1 | 46,2 |
| *iniA* | 1,9 | 32,7 | 5,6 |
| *iniC* | 1,3 | 7,9 | 1,9 |
