# Supplementary material for: Cell envelope stress in mycobacteria is regulated by the novel signal transduction ATPase IniR in response to trehalose
Source: PLoS Genet. 2017 Dec 27;13(12):e1007131. doi: 10.1371/journal.pgen.1007131 (PMC5760070; doi:10.1371/journal.pgen.1007131)
Supplement: S3 Table — (DOCX) [file pgen.1007131.s010.docx]

S3 Table

| Primer name | Sequence 5’ 🡪 3’ |
| --- | --- |
| iniR_Mm-_Comp_FW | AGGTTAATTAAAGCTTCATCGGCGATAGCCTGCG |
| iniR_Mm_-Comp_RV | CGACATCGATAAGCTTTCAGCCCGGTGCCG |
| iniR_Mm_-FLAG_FW | ATGACAAGTTTGTACAAAAAAGCAGGCTCTGTGTCCGAGGGCCACGCTTC |
| iniR_Mm_-FLAG_RV | TCTACGACTTTGTACAAGAAAGCTGAGTCGCCCGGTGCCGTCAACATCGC |
| iniR_Mm__L-FW | GACTCACTCCAAAAACGACCGGCGCTCTGCCATAA |
| iniR_Mm_ _L-RV | TACAGGACCTGCCAATACCCCATCTCGCTCTCCCCG |
| iniR_Mm_ _R-FW | GACTCTAGCCAAAGACTGGCCGACCAGGTGGATGT |
| iniR_Mm_ _R-RV | TGTCAAACCTGCCAACCGCTGTTGTGCGAGCCATA |
| iniR_Mtb_-Strep-FW | TGTACAGTACAAAAAAGCAGGCTCTATGCAGC |
| iniR_Mtb_-Strep-RV | TGTACATTACTTCTCGAACTGCGGGTGGCTCCATCCGCTTCCTCCGCTTCCACGCCTCTCATCTGCGGTTA |
| iniR_Mtb_-BsrGI-FW | ATGACAAGTTTGTACATGTACAGTACAAAAAAGCAGGCTCTATG |
| iniR_Mtb_-BsrGI-RV | TCTACGACTTTGTACATGTACATTACTTCTCGAACTGCGG |
