# Supplementary material for: Cell envelope stress in mycobacteria is regulated by the novel signal transduction ATPase IniR in response to trehalose
Source: PLoS Genet. 2017 Dec 27;13(12):e1007131. doi: 10.1371/journal.pgen.1007131 (PMC5760070; doi:10.1371/journal.pgen.1007131)
Supplement: S4 Table — (DOCX) [file pgen.1007131.s011.docx]

S4 Table

| **Name** | **Features** | **Source** |
| --- | --- | --- |
| pSMT3*-piniB4-mEos3.1* | *iniB* promoter, *mEos3.1*, *hyg*^R^ | [13] |
| pSMT3*-hsp60-mspA* | *hsp60* promoter and *mspA*, *hyg*^R^ | [20] |
| pEXCF-*iniR_Mtb_*-*FLAG* | *tetR/tetO*, (*Rv0339c*) *iniR_Mtb_*-*FLAG*, *hyg*^R^ | [40] |
| pEXCF-*iniR_Mm_-FLAG* | *tetR/tetO*, (*MMAR_0612*) *iniR_Mm_*-*FLAG*, *hyg*^R^ | This study |
| pEXCF*-iniR_Mtb_-Strep* | *tetR/tetO, iniR_Mtb_*-*Strep, hyg*^R^ | This study |
| pMV-*piniB4-mEos3.1* | *iniB* promoter, *mEos3.1*, L5 *attP* & *aph, oriE, str*^R^ | [13] |
| pMV-*noHSP* | L5 *attP* & *aph, oriE, str*^R^ | [13] |
| pMV-pr*iniR_Mm_* | *iniR* promoter, *iniR_Mm_*  (*MMAR_0612*), L5 *attP* & *aph, oriE, str*^R^ | This study |
