# Supplementary material for: Cell envelope stress in mycobacteria is regulated by the novel signal transduction ATPase IniR in response to trehalose
Source: PLoS Genet. 2017 Dec 27;13(12):e1007131. doi: 10.1371/journal.pgen.1007131 (PMC5760070; doi:10.1371/journal.pgen.1007131)
Supplement: S5 Table — (DOCX) [file pgen.1007131.s012.docx]

S5 Table

| **Name** | **Strain** | **Features/Mutation(s)** | **Source** |
| --- | --- | --- | --- |
| WT *M. marinum* | *M. marinum* M^USA^ | - | [37] |
| WT *M. smegmatis* | *M. smegmatis* MC^2^155 | - | W.R. Jacobs |
| WT *M. tuberculosis* | *M. tuberculosis* H37Rv |  |  |
| *cobC::tn* | *M. marinum* M^USA^ | Transposon insertion in *cobC* (*MMAR_3307* bp 273) | [13] |
| ∆*iniR_Mm_* | *M. marinum* M^USA^ | Deletion of *MMAR_0612* | This study |
| ∆*iniR_Mtb_* | *M. tuberculosis* H37Rv | Deletion of *Rv0339c* | This study |
| *sugA::tn* | *M. marinum* M^USA^ | Transposon insertion in *sugA* (*MMAR_4205* bp 336) | This study |
